# A Dual‐Programmable 2M NOR Flash Architecture for Energy‐Efficient In‐Memory Computing

**DOI:** 10.1002/advs.77797

**Published:** 2026-09-27

**Authors:** Suhan Kim, Donghyun Ryu, Hyoseob Kim, Sangwoo Jung, Min‐Hwi Kim

**Affiliations:** ^1^ Department of Intelligent Semiconductor Engineering Chung‐Ang University Seoul Republic of Korea; ^2^ Department of Electrical Engineering and Computer Sciences University of California Berkeley USA; ^3^ School of Electrical and Electronics Engineering Chung‐Ang University Seoul Republic of Korea

**Keywords:** charge‐trap synaptic device, compute‐in‐memory, energy‐efficient computing, hardware pruning, neural network inference, NOR Flash memory

## Abstract

Conventional NOR Flash architectures employ either a 1M structure or a 1T–1M structure, the latter integrating an access transistor to enable selective program and erase operations. While the access transistor occupies a substantial portion of the 1T–1M cell area, it does not contribute to information storage. Here, a 2M NOR Flash architecture is demonstrated in which two series‐connected charge‐trap transistors (${\mathrm{Cell}}_{1}$ and ${\mathrm{Cell}}_{2}$) simultaneously function as programmable memory elements and current‐controlling components, enabling conductance‐window extension and current‐path gating within a single device structure. Device measurements show that sequential programming of the two memory devices significantly extends the accessible current window, reducing the minimum read current from 72.7 nA to 4.22 pA while maintaining reliable inhibit behavior under array bias conditions. Hardware‐aware neural inference further demonstrates that the extended conductance window reduces inference power by approximately 45% across multiple neural networks. In addition, the second programmable memory device enables true‐off hardware pruning and ${\mathrm{Cell}}_{2}$ state modulation, allowing substantial reduction of inference power while maintaining classification accuracy. These dual functional roles establish a task‐adaptive operation framework, in which the proposed 2M NOR Flash architecture serves as a scalable device platform for energy‐efficient compute‐in‐memory systems.

## Graphical abstract

In conventional NOR Flash, the access transistor occupies area but stores no information. Here, it is repurposed as a programmable second memory device that plays two functional roles: an analog memory element that lowers the minimum read current from 72.7 nA to 4.22 pA, and a current‐path gating element enabling true‐off pruning and current‐range modulation for energy‐efficient compute‐in‐memory.

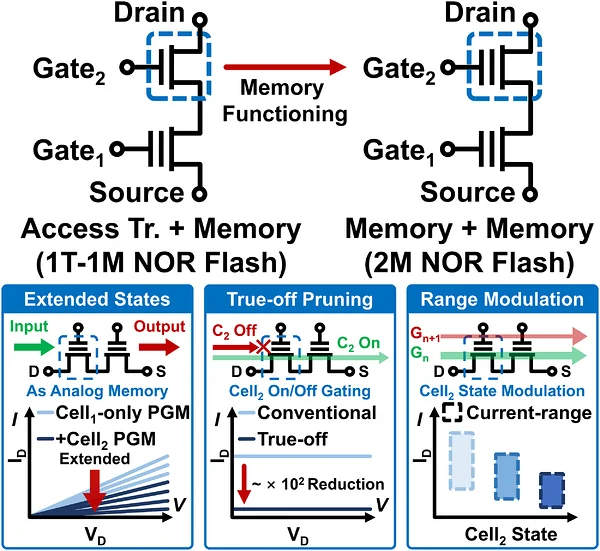

## 1 Introduction

The growing demand for energy‐efficient artificial intelligence hardware has stimulated extensive research into compute‐in‐memory (CIM) architectures capable of performing analog multiply–accumulate (MAC) operations directly within memory arrays [1, 2, 3]. Various emerging and conventional non‐volatile memory technologies, including resistive random‐access memory (RRAM), phase‐change memory (PCM), ferroelectric RAM (FeRAM), magnetic RAM (MRAM), and Flash memory, have been explored as synaptic devices for neuromorphic and CIM systems [4, 5, 6, 7, 8, 9, 10, 11, 12].

Among these candidate technologies, Flash memory is particularly attractive for CIM applications because of its high integration density, mature CMOS‐compatible fabrication infrastructure, and compatibility with large‐scale memory manufacturing [13, 14, 15]. Recent demonstrations of Flash‐based or Flash‐derived neuromorphic and in‐memory computing hardware further highlight the potential of this technology family for energy‐efficient neural computing [16, 17, 18, 19, 20, 21].

The suitability of Flash memory for analog computing is strongly influenced by the underlying program and erase mechanisms that determine charge storage behavior and device reliability. In Flash devices, program and erase operations are typically achieved through hot‐carrier injection (HCI) or Fowler–Nordheim (FN) tunneling [15, 22, 23, 24]. HCI‐based programming relies on high‐energy carriers, which can induce localized oxide damage and spatially non‐uniform charge trapping, whereas FN tunneling enables more uniform charge transfer across the oxide and is therefore attractive for reliable operation in Flash‐based computing devices [22, 23, 24].

To support selective operation under such erase conditions, practical Flash arrays generally require a cell structure that electrically isolates the memory device from unselected channel regions [15, 25, 26]. In conventional NOR Flash architectures, this requirement is commonly addressed by adopting a 1T–1M structure, in which an access transistor is integrated alongside the memory device [15, 26, 27]. While simpler 1M configurations exist, the 1T–1M structure is widely used in practical NOR Flash arrays for its selectivity under array bias conditions. Recent Flash‐memory developments, including advanced NOR‐compatible architectures, highly integrated Flash chips, and Flash‐based vector–matrix operation, further indicate that array compatibility, density, and update controllability remain central design constraints in practical hardware implementations [26, 28, 29, 30]. Despite its essential role in enabling selective operation, however, the access transistor itself does not participate in information storage. Consequently, a substantial portion of the cell structure in 1T–1M NOR architectures is assigned to a component that does not directly contribute to memory density or computational functionality.

To address this limitation, a 2M NOR Flash architecture is introduced in which the access transistor of the conventional 1T–1M cell is repurposed as a second programmable charge‐trap memory device (${\mathrm{Cell}}_{2}$). In this configuration, ${\mathrm{Cell}}_{2}$ plays two distinct functional roles depending on its programmed state: it can serve as an additional analog memory element that, in series with ${\mathrm{Cell}}_{1}$, extends the effective conductance window, or as a current‐path gating element that programmably blocks or restricts the current flow through the device.

**FIGURE 1:**
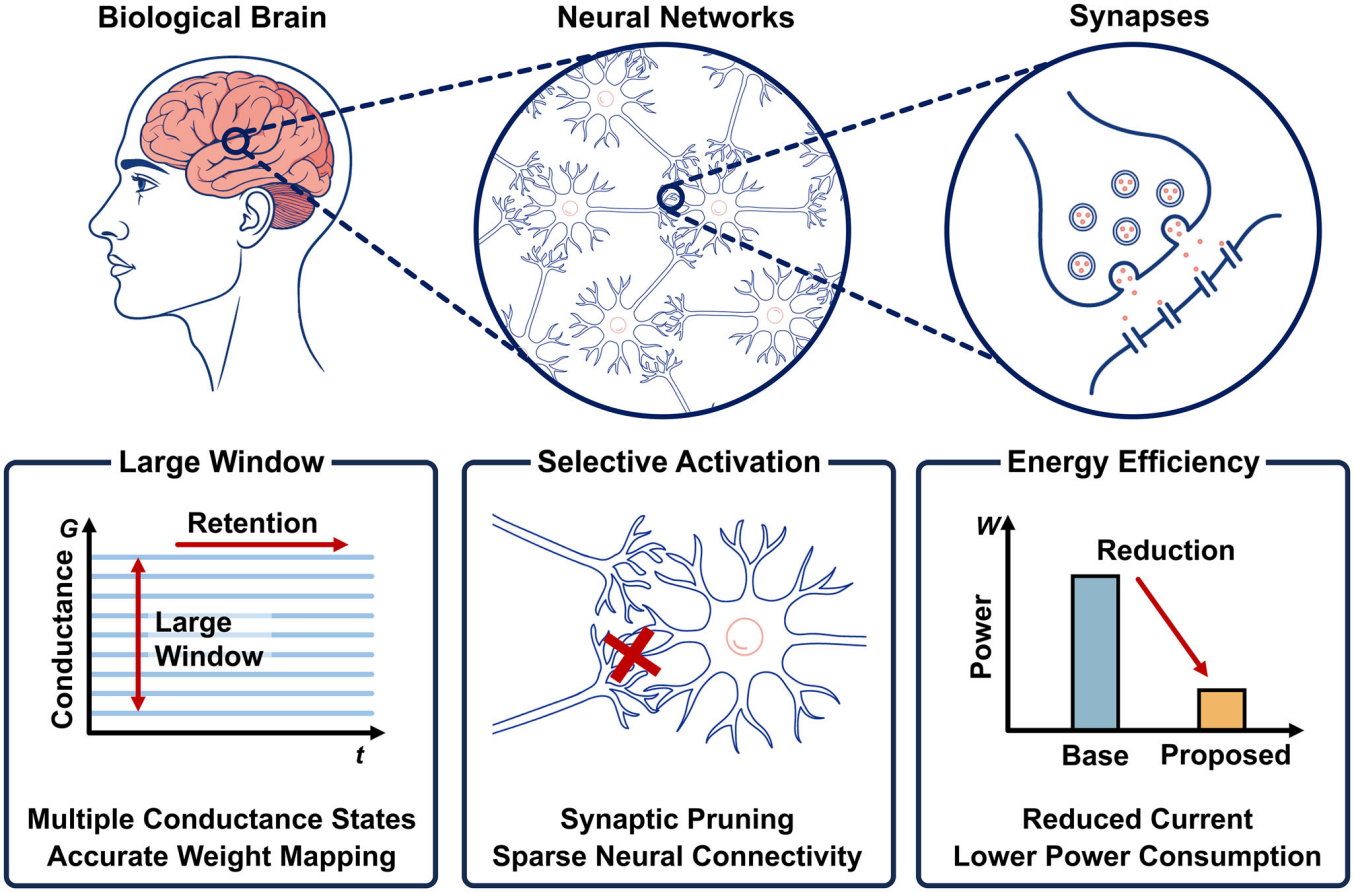
Key requirements for synaptic memory devices in neuromorphic computing systems. Conceptual illustration of the requirements for synaptic devices, including a wide analog conductance window, selective activation of synaptic connections, and energy‐efficient operation.

Analog in‐memory computing hardware has shown strong potential for efficient neural inference, but its practical performance remains constrained by limited conductance range, residual current, and energy consumption during array‐level operation [31, 32, 33]. For energy‐efficient neuromorphic computing, an ideal synaptic memory device should simultaneously provide a wide analog conductance window, selective activation of synaptic connections, and low‐power operation, as conceptually summarized in Figure 1 [34, 35, 36]. However, these requirements are difficult to satisfy simultaneously in conventional NOR Flash architectures due to the limited functionality of the access transistor and the restricted conductance range of a single memory device. In this work, it is experimentally demonstrated that the proposed 2M NOR Flash device enables both analog conductance extension and programmable current‐path gating within a single device structure. Device measurements show that sequential programming of two memory devices extends the accessible current window from the nA range to the pA range, while the programmable state of the second memory device enables hardware‐level pruning and current‐range modulation. Through inhibit verification under array bias conditions and hardware‐aware neural inference, the architecture is further confirmed to maintain reliable selective operation while significantly reducing inference power. Importantly, the proposed structure remains fully compatible with conventional FN‐based NOR Flash fabrication flows and does not require additional masks or fundamentally new process modules.

## 2 Results and Discussion

### 2.1 Architecture and Operating Principle of the Proposed 2M NOR Flash Device

**FIGURE 2:**
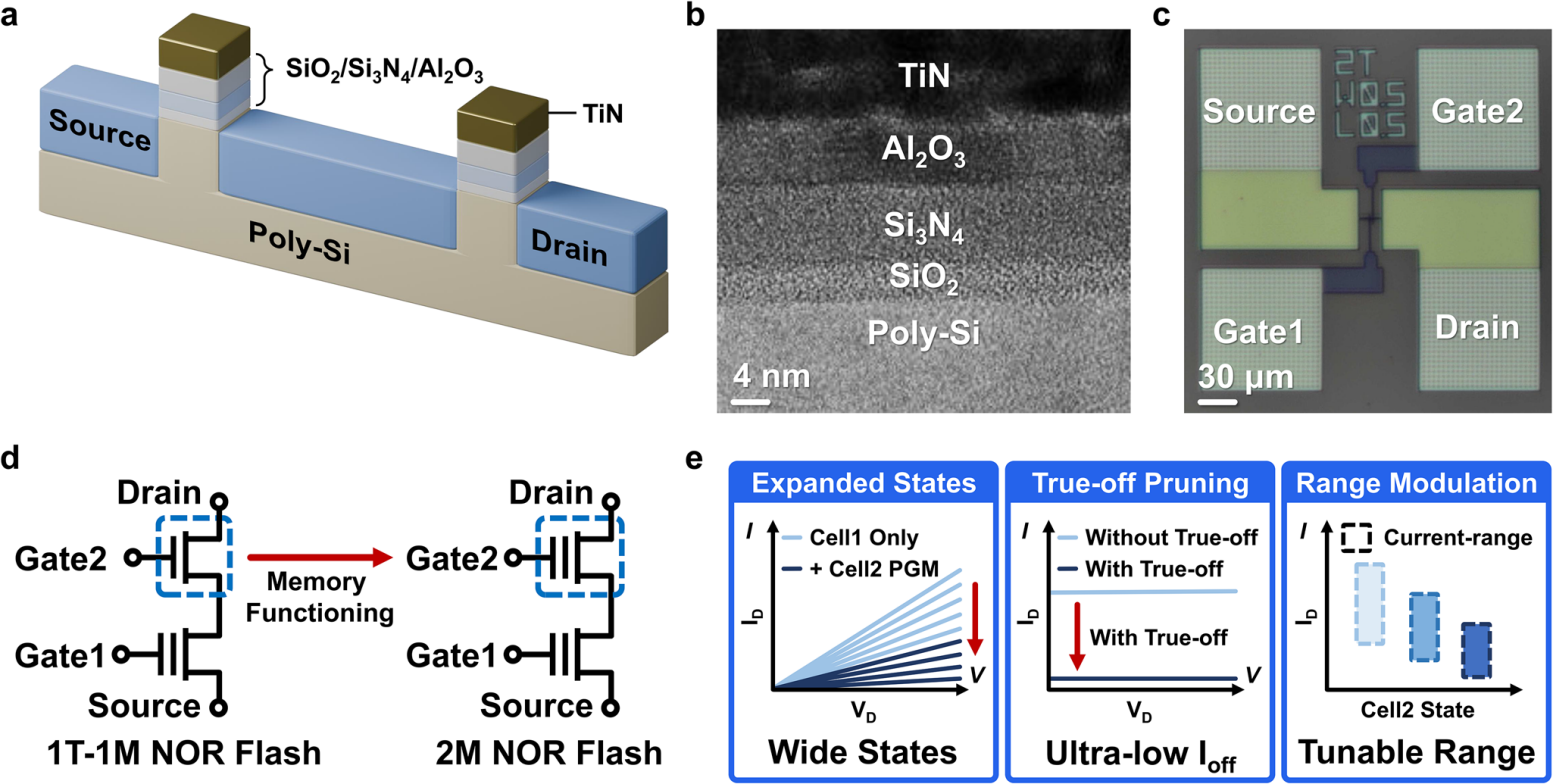
Device structure, conceptual transformation, and ${\mathrm{Cell}}_{2}$ functional roles of the proposed 2M NOR Flash architecture. (a) Schematic illustration of the proposed 2M NOR Flash device structure. (b) Cross‐sectional transmission electron microscopy image of a charge‐trap memory device, showing the TiN/${\mathrm{Al}}_{2}O_{3}$/${\mathrm{Si}}_{3}N_{4}$/${\mathrm{SiO}}_{2}$/poly‐Si stack. (c) Optical top‐view image of the fabricated device, showing the source, drain, ${\mathrm{Gate}}_{1}$, and ${\mathrm{Gate}}_{2}$ terminals. (d) Conceptual comparison between the conventional 1T–1M NOR Flash cell and the proposed 2M NOR Flash cell, illustrating the transformation of the access transistor into an additional programmable memory element. (e) Two functional roles of ${\mathrm{Cell}}_{2}$ in the proposed 2M NOR Flash architecture: as an analog memory element enabling conductance‐window extension, and as a current‐path gating element enabling current suppression and state modulation.

The architecture of the proposed 2M NOR Flash device is described first (Figure 2a). The charge‐trap gate stack used in this work [37] is shown in the cross‐sectional TEM image of the charge‐trap memory device in Figure 2b, and the layout of the manufactured device with independently accessible source, drain, ${\mathrm{Gate}}_{1}$ and ${\mathrm{Gate}}_{2}$ terminals is presented in the optical top‐view image in Figure 2c.

In contrast to conventional 1T–1M NOR structures, where only one device stores information and the other serves as a passive access transistor, the proposed structure allows the access transistor to also function as a programmable memory element. As conceptually illustrated in Figure 2d, this transformation converts the conventional 1T–1M configuration into a 2M NOR Flash architecture in which both devices operate as memory elements. Because the two memory devices are connected in series, the effective conductance of the 2M NOR Flash device is jointly determined by their programmed states, enabling mutual modulation of the overall current.[Supplementary-material advs77797-supl-0001]

The two functional roles of ${\mathrm{Cell}}_{2}$ are illustrated in Figure 2e. When ${\mathrm{Cell}}_{2}$ is programmed into analog states together with ${\mathrm{Cell}}_{1}$, it serves as an additional analog memory element whose conductance is combined with that of ${\mathrm{Cell}}_{1}$ through the series configuration, enabling an extended effective conductance window. Alternatively, when ${\mathrm{Cell}}_{2}$ is programmed to strongly suppress the current path, it acts as a current‐path gating element that enables explicit control of synaptic activation, including complete suppression of current for pruning and selective modulation of the effective current range. Through these two functional roles of ${\mathrm{Cell}}_{2}$, the proposed 2M NOR Flash device unifies conductance extension and programmable current‐path gating within a single device structure.

### 2.2 Access Transistor as an Analog Memory Element

**FIGURE 3:**
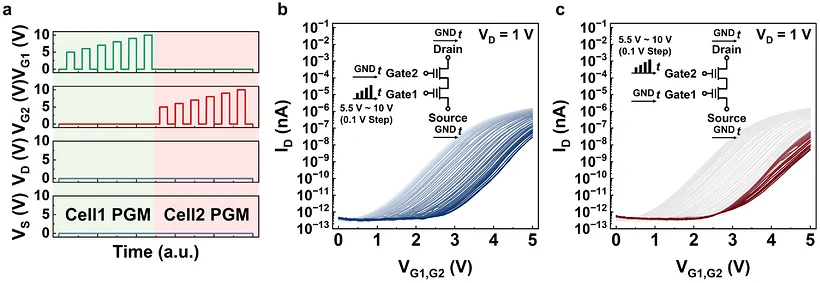
${\mathrm{Cell}}_{2}$ as an analog memory element in the proposed 2M NOR Flash device. (a) Sequential programming scheme of the 2M NOR Flash device. (b) Transfer characteristics ($I_{D}$–$V_{G}$) under ${\mathrm{Cell}}_{1}$ programming, showing the formation of multiple analog states. (c) Transfer characteristics ($I_{D}$–$V_{G}$) under additional ${\mathrm{Cell}}_{2}$ programming, showing further modulation of the device characteristics.

When ${\mathrm{Cell}}_{2}$ is utilized as an additional analog memory element, the effective conductance of the 2M NOR Flash device is jointly determined by the analog states of ${\mathrm{Cell}}_{1}$ and ${\mathrm{Cell}}_{2}$, as illustrated in Figure 3. The operation is based on a sequential programming scheme, as shown in Figure 3a, in which ${\mathrm{Cell}}_{1}$ and ${\mathrm{Cell}}_{2}$ are programmed in sequence.

Figure 3b shows the transfer characteristics obtained after programming ${\mathrm{Cell}}_{1}$. As the programming condition is gradually varied, multiple analog states are formed, establishing the base states of the device.

After defining the states of ${\mathrm{Cell}}_{1}$, additional programming of ${\mathrm{Cell}}_{2}$ further modulates the device characteristics. As shown in Figure 3c, the measured $I_{D}$–$V_{G}$ curves shift systematically after ${\mathrm{Cell}}_{2}$ programming, indicating that the second memory device introduces an additional degree of modulation.

Because ${\mathrm{Cell}}_{2}$ modulation is applied on top of the states defined by ${\mathrm{Cell}}_{1}$, the effective electrical characteristics of the device are jointly determined by the programmed states of both memory devices. This sequential programming scheme therefore extends the accessible state space beyond that of a single‐device implementation.

### 2.3 Drain‐Input Encoding for Analog MAC Operation

The proposed architecture enables MAC operation through a drain‐input encoding scheme, in which the input signal is applied to the drain terminal while the programmed conductance of the 2M NOR Flash device represents the synaptic weight. In this configuration, the output current in the low drain‐voltage regime approximately follows the relationship given in Equation (1):

(1) $$I_{D}\propto G_{\mathrm{device}}V_{D}.$$

As described by Equation (1), the drain current is jointly determined by the device conductance and the input voltage.

**FIGURE 4:**
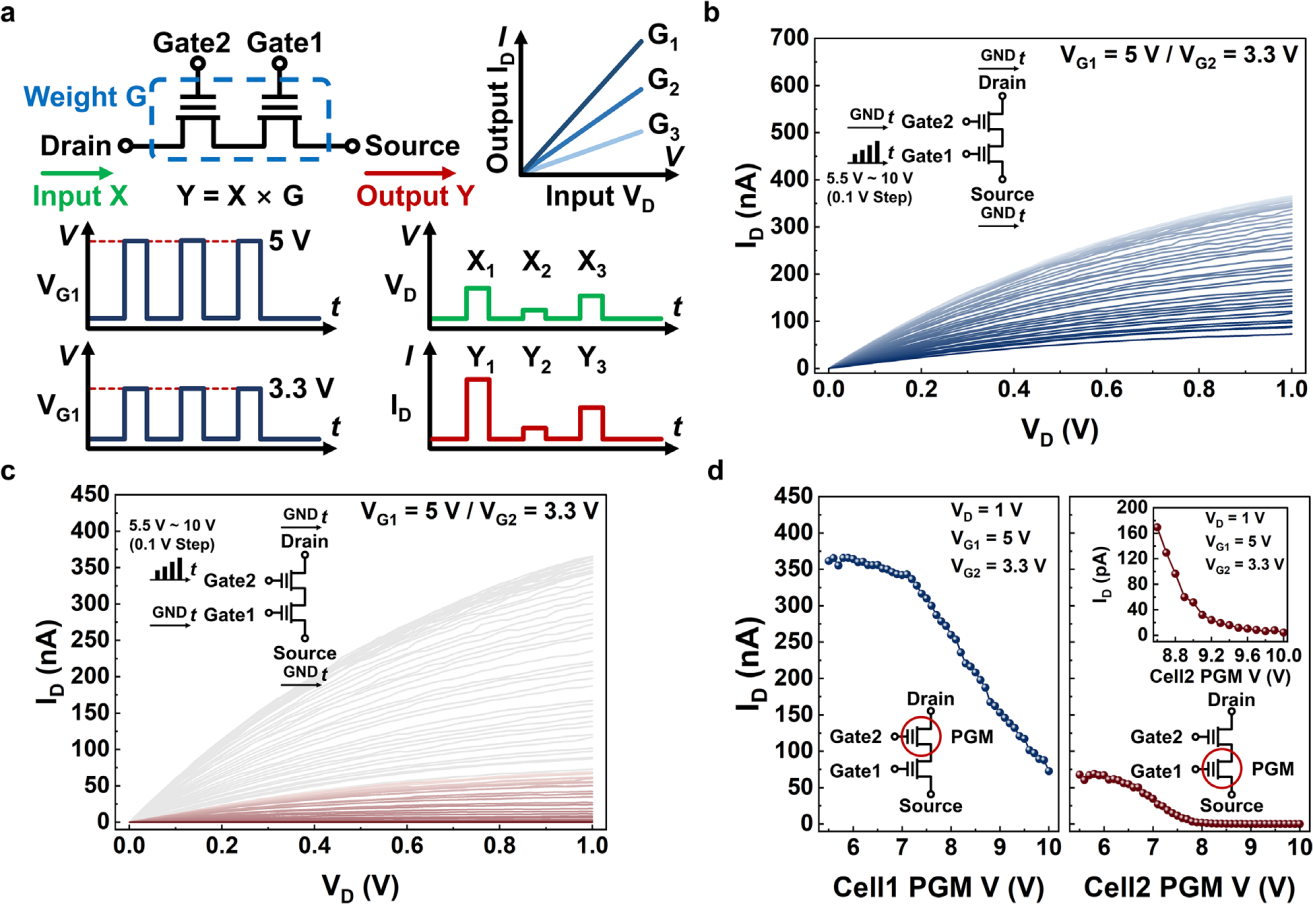
Drain‐input encoding for analog multiply–accumulate operation. (a) Schematic illustration of the drain‐input encoding scheme, where the drain voltage represents the input signal and the programmed state of the 2M NOR Flash device represents the synaptic weight. (b) Output characteristics ($I_{D}$–$V_{D}$) under ${\mathrm{Cell}}_{1}$ programming, defining the baseline current range of the device. (c) Output characteristics ($I_{D}$–$V_{D}$) under additional ${\mathrm{Cell}}_{2}$ programming on top of the states defined by ${\mathrm{Cell}}_{1}$, showing systematic modulation toward lower current levels. (d) Distribution of read currents, showing the reduction of the minimum read current from 72.7 nA to 4.22 pA.

Figure 4a illustrates the drain‐input encoding scheme used in this work. The drain voltage represents the input signal, while the programmed conductance state of the 2M NOR Flash device determines the effective synaptic weight.

Figure 4b shows the measured $I_{D}$–$V_{D}$ characteristics when only ${\mathrm{Cell}}_{1}$ is programmed, defining the baseline current range of the device. In this case, the achievable current range is limited by the states of a single memory device.

Figure 4c shows the $I_{D}$–$V_{D}$ characteristics after additional programming of ${\mathrm{Cell}}_{2}$ on top of the pre‐defined ${\mathrm{Cell}}_{1}$ states. The current characteristics shift systematically toward lower current levels over the entire voltage range, indicating that the second memory device provides additional control over the current path.

The resulting read current distribution is summarized in Figure 4d. The minimum read current is reduced from 72.7 nA to 4.22 pA through sequential programming of the two memory devices. This reduction is particularly important for CIM systems, because analog MAC operation in memory arrays is directly governed by device current and voltage, and lower‐current operation provides an effective route toward reducing inference power in hardware‐aware in‐memory computing systems.

### 2.4 Access Transistor as a Current‐Path Gating Element

**FIGURE 5:**
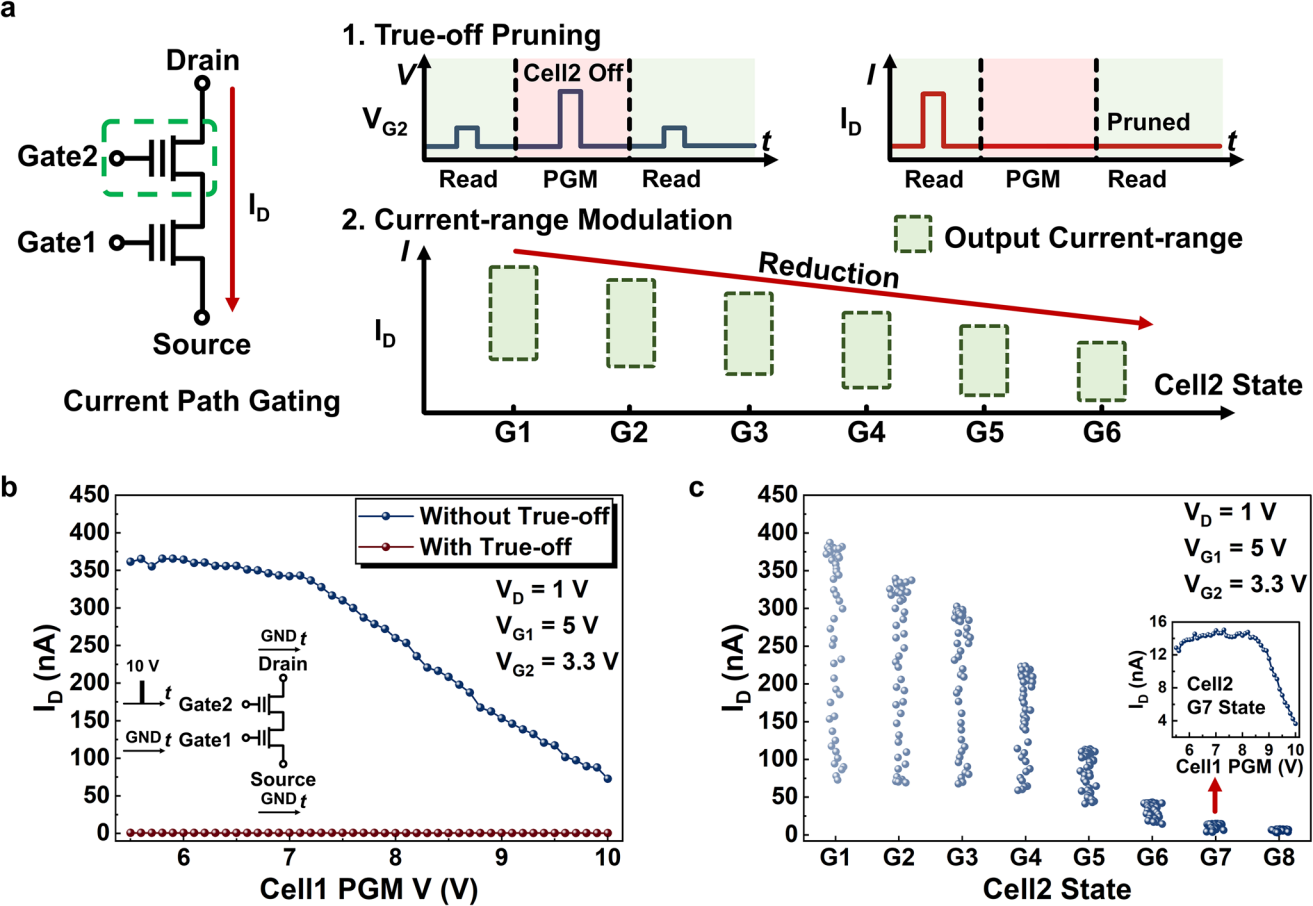
${\mathrm{Cell}}_{2}$ as a current‐path gating element in the proposed 2M NOR Flash device. (a) Conceptual illustration of current‐path gating enabled by the programmable state of ${\mathrm{Cell}}_{2}$, including true‐off pruning and current‐range modulation. (b) Experimental verification of the ${\mathrm{Cell}}_{2}$ OFF state, showing that the current path can be effectively suppressed regardless of the conductance state of ${\mathrm{Cell}}_{1}$. (c) Modulation of the output current range using intermediate states of ${\mathrm{Cell}}_{2}$. Note that each distribution represents the accessible current range attainable by ${\mathrm{Cell}}_{1}$ programming under the corresponding ${\mathrm{Cell}}_{2}$ state, rather than the statistical spread of a single conductance level. A specific ${\mathrm{Cell}}_{2}$ state is selected according to the target task.

Beyond its role as an analog memory element, ${\mathrm{Cell}}_{2}$ can also function as a current‐path gating element that programmably controls the current flow through the device. As conceptually illustrated in Figure 5a, the programmable state of ${\mathrm{Cell}}_{2}$ can be used either to strongly suppress the current path or to modulate the accessible current range.

When ${\mathrm{Cell}}_{2}$ is programmed into the OFF state, the current path of the 2M NOR Flash device is effectively blocked regardless of the conductance state stored in ${\mathrm{Cell}}_{1}$. Figure 5b experimentally verifies this behavior, showing that the device current is almost completely suppressed. This functionality enables hardware‐level control of synaptic activation, providing true‐off pruning that completely suppresses the current path, unlike conventional analog CIM approaches where pruned weights still produce residual current.

Such cell‐wise permanent suppression cannot be achieved in a single‐memory‐device implementation, in which even the most deeply programmed cell still conducts a residual current of 72.7 nA. Gate‐bias‐based deactivation of the access transistor is likewise limited, because the shared word line turns off an entire row and the bias‐driven off state is volatile, whereas the ${\mathrm{Cell}}_{2}$ OFF state is stored in the charge‐trap layer and is therefore cell‐wise independent and non‐volatile.

Alternatively, intermediate conductance states of ${\mathrm{Cell}}_{2}$ allow the effective current range of the device to be modulated. As shown in Figure 5c, the current distribution shifts systematically toward lower values as the programmed state of ${\mathrm{Cell}}_{2}$ changes. This behavior originates from the additional series resistance introduced by ${\mathrm{Cell}}_{2}$, which limits the overall current flowing through the device.

These results show that ${\mathrm{Cell}}_{2}$ serves as a programmable current‐path controller, enabling both true‐off synaptic gating and current‐range modulation within a single device.

### 2.5 Inhibit Validation Under Array Operating Conditions

To evaluate the feasibility of selective operation in array environments, inhibit behavior was investigated by applying array‐relevant bias conditions to fabricated devices.

**FIGURE 6:**
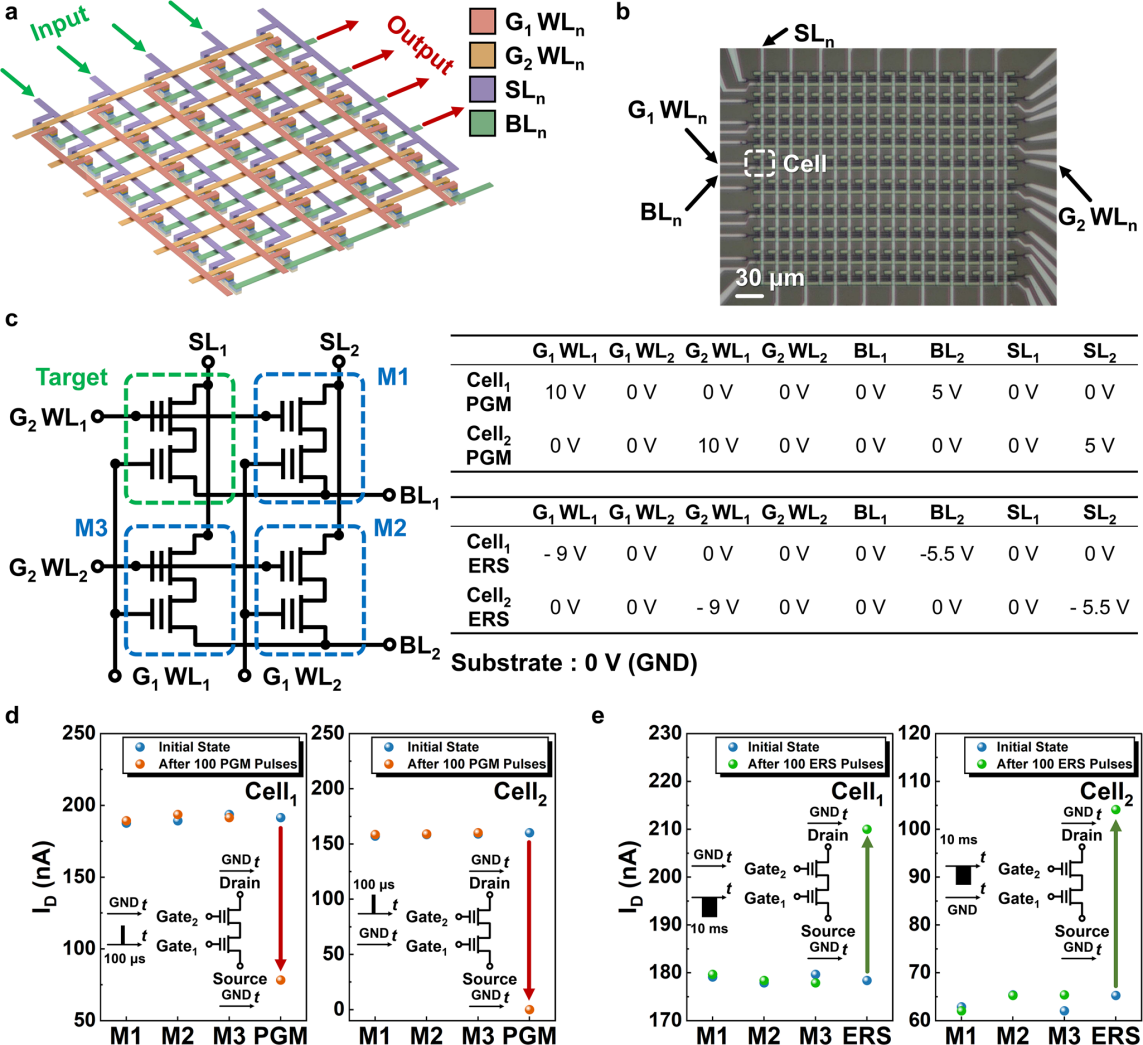
Inhibit validation under array‐relevant operating conditions. (a) Schematic illustration of the fabricated NOR Flash array. (b) Optical micrograph of the fabricated NOR Flash array. (c) Array operation scheme and bias configuration used for the inhibit evaluation. The table summarizes the bias conditions applied during program and erase operations, and the substrate is held at 0 V (grounded) for all operating modes. (d) Program inhibit evaluation for both ${\mathrm{Cell}}_{1}$ and ${\mathrm{Cell}}_{2}$ programming operations under array‐relevant bias conditions, showing that the selected device is properly programmed while inhibited devices remain stable. (e) Erase inhibit evaluation for both ${\mathrm{Cell}}_{1}$ and ${\mathrm{Cell}}_{2}$ erase operations under array‐relevant bias conditions, showing that the selected device is properly erased while inhibited devices remain stable.

Figure 6a,b show the structure and optical image of the fabricated NOR Flash array used in this study. The array consists of ${\mathrm{Gate}}_{1}$ word lines ($G_{1}\mathrm{WL}$), ${\mathrm{Gate}}_{2}$ word lines ($G_{2}\mathrm{WL}$), bit lines (BL), and source lines (SL), enabling selective addressing of individual devices.

The biasing scheme used for the disturb evaluation is illustrated in Figure 6c. In this configuration, the target device is programmed or erased while neighboring devices sharing the same $G_{1}\mathrm{WL}$, $G_{2}\mathrm{WL}$, BL, or SL are biased under inhibit conditions to prevent unintended state changes. The detailed bias conditions used in these experiments are summarized in the table of Figure 6c.

Program inhibit behavior was evaluated for both ${\mathrm{Cell}}_{1}$ and ${\mathrm{Cell}}_{2}$ programming operations. As shown in Figure 6d, the selected device exhibits the intended programming‐induced current change, while the inhibited devices remain stable under the corresponding bias conditions. This confirms that program disturb is effectively suppressed for both ${\mathrm{Cell}}_{1}$ and ${\mathrm{Cell}}_{2}$ programming operations.

Erase inhibit behavior was also investigated for both ${\mathrm{Cell}}_{1}$ and ${\mathrm{Cell}}_{2}$ erase operations. As shown in Figure 6e, the selected device exhibits the expected erase‐induced current change, whereas the inhibited devices remain nearly unchanged. These results confirm that erase disturb is effectively suppressed for both ${\mathrm{Cell}}_{1}$ and ${\mathrm{Cell}}_{2}$ erase operations.

These results indicate that the proposed 2M NOR Flash architecture maintains reliable inhibit behavior under array operating conditions.

### 2.6 System‐Level Validation for Compute‐in‐Memory Inference

To evaluate the system‐level implications of the proposed architecture, hardware‐aware neural inference was performed using both compact‐model‐based simulations and measured lookup table data. Hardware‐aware evaluation is important in in‐memory computing systems because device characteristics and current–voltage nonidealities can directly influence inference accuracy and energy efficiency.

Detailed training configurations, including network architectures, optimization settings, and training procedures, are provided in the Supporting Information.

In this framework, input data from CIFAR‐10 and MNIST are first processed through a feature extractor or directly normalized, and then applied to a single fully connected layer. The resulting feature vectors are converted into input voltages and mapped to the proposed 2M NOR Flash array using the drain‐input encoding scheme. The synaptic weights are represented by the programmed conductance states of the memory devices, and the corresponding drain currents are computed using either measured lookup table data or the compact model.

**FIGURE 7:**
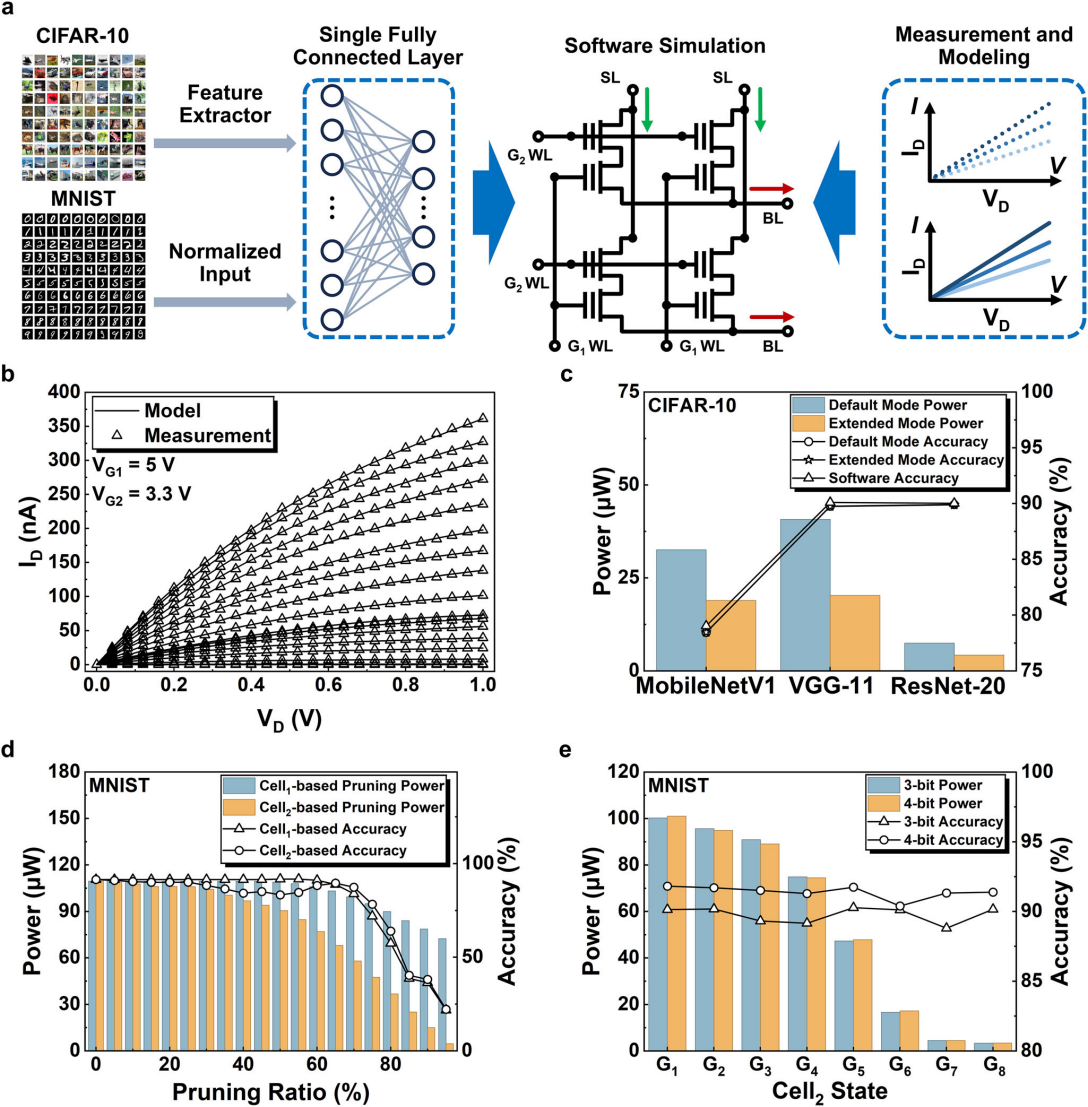
System‐level validation of the proposed architecture for compute‐in‐memory inference. (a) Hardware‐aware inference pipeline. CIFAR‐10 and MNIST inputs are processed by a feature extractor or directly normalized, followed by a single fully connected layer and mapped to the proposed 2M NOR Flash array using drain‐input encoding with measured lookup table data or compact modeling. (b) Comparison between measured lookup table and compact‐model‐reconstructed current characteristics, confirming accurate reproduction across conductance states. (c) Hardware inference accuracy and average inference power for CIFAR‐10 using MobileNetV1, VGG‐11, and ResNet‐20, comparing the default mode and extended mode. (d) Comparison of pruning implementations for MNIST, where pruned weights are mapped either to the minimum conductance state or to the ${\mathrm{Cell}}_{2}$ OFF state. (e) Inference power and classification accuracy for MNIST as functions of ${\mathrm{Cell}}_{2}$ state, evaluated for 3‐bit and 4‐bit weights.

Figure 7b compares the current characteristics reconstructed from the compact model with the measured lookup table data, confirming that the model accurately reproduces the device behavior across conductance states. For CIFAR‐10 classification, the feature extractor portions of convolutional neural networks, including MobileNetV1, VGG‐11, and ResNet‐20, were used. The extracted features were then processed by a single fully connected layer, whose trained weights were mapped to the conductance states of the proposed device model using the compact model. Figure 7c summarizes the hardware inference accuracy and the average inference power for CIFAR‐10 across multiple neural network architectures. Here, the default mode refers to the conductance range obtained using a single memory device, whereas the extended mode corresponds to the conductance‐window extension enabled by the dual‐programmable 2M architecture. Compared with the default mode based on a single memory device, the extended mode enabled by the proposed architecture maintains classification accuracy while reducing the inference power by approximately 41.7%–50.2% (with an average reduction of approximately 45%). This reduction originates from the ability to access lower current levels during analog MAC operations, which directly decreases the current‐driven energy consumption in the array.

To investigate the role of ${\mathrm{Cell}}_{2}$ as a current‐path gating element, additional simulations were performed on the MNIST classification task using directly measured lookup table data. Figure 7d compares two pruning strategies in which pruned weights are mapped to the minimum conductance state or completely suppressed using the ${\mathrm{Cell}}_{2}$ OFF state. The ${\mathrm{Cell}}_{2}$‐based pruning configuration suppresses unnecessary current paths more effectively while maintaining comparable classification accuracy. As a result, the inference power is reduced from 95 to 47.5 μW at a pruning ratio of 75%, corresponding to approximately 50% reduction in power consumption.

Finally, the impact of ${\mathrm{Cell}}_{2}$ state on inference performance was evaluated for the MNIST classification task. As shown in Figure 7e, the inference power and classification accuracy were analyzed as functions of ${\mathrm{Cell}}_{2}$ state for both 3‐bit and 4‐bit weights. As the ${\mathrm{Cell}}_{2}$ state increases, the inference power decreases substantially, while the classification accuracy remains around 90% for both cases. In particular, for the 4‐bit case, the inference power decreases from 101 μW at ${\mathrm{Cell}}_{2}$ state 1 to 3.39 μW at ${\mathrm{Cell}}_{2}$ state 8 while maintaining a classification accuracy of approximately 91%.

These results indicate that, for low‐complexity tasks such as MNIST, the synaptic current range can be deliberately compressed to reduce power consumption while preserving inference accuracy.

## 3 Conclusion

In this work, it is shown that extending the role of the conventional access transistor beyond passive selection enables a 2M NOR Flash architecture with both an extended conductance window and programmable current‐path gating. This dual functionality broadens the capability of NOR Flash from conventional memory operation toward energy‐efficient compute‐in‐memory applications.

A central advantage of the proposed 2M NOR Flash device is the significant extension of the accessible current window through sequential programming of two memory devices. Experimental measurements showed that programming of ${\mathrm{Cell}}_{2}$ substantially extended the low‐current regime, reducing the minimum read current from 72.7 nA to 4.22 pA. This extended low‐current accessibility is particularly beneficial for analog CIM systems, because the energy consumption of MAC operations scales directly with the read current. By enabling synaptic weights to be represented at much lower operating currents, the proposed architecture provides an effective device‐level route toward reducing inference power.

Beyond conductance‐window extension, ${\mathrm{Cell}}_{2}$ also functions as a programmable current‐path gating element within the same structure. When programmed into a strongly suppressed state, ${\mathrm{Cell}}_{2}$ effectively blocks the current path of the synapse, enabling true‐off hardware pruning that removes unnecessary current flow during inference. This behavior differs fundamentally from conventional analog CIM pruning approaches, in which pruned weights are mapped only to the minimum conductance state and still generate residual current. In addition, intermediate ${\mathrm{Cell}}_{2}$ states provide a simple means to modulate the effective current range of the device, allowing flexible trade‐offs between inference accuracy and energy consumption.

System‐level evaluation further confirmed the practical benefits of the proposed architecture. Hardware‐aware inference on CIFAR‐10 showed that the extended conductance window enables stable neural inference while reducing the average inference power across multiple neural network architectures. In lookup‐table‐based MNIST evaluation, ${\mathrm{Cell}}_{2}$‐assisted pruning effectively suppressed redundant current paths while preserving classification accuracy. Furthermore, ${\mathrm{Cell}}_{2}$ state modulation reduced the inference power from 101 to 3.39 μW while maintaining an accuracy of approximately 91%, demonstrating that the synaptic current range can be intentionally compressed to minimize energy consumption for low‐complexity inference tasks.

Overall, the proposed 2M NOR Flash architecture establishes a task‐adaptive operation framework in which the two functional roles of ${\mathrm{Cell}}_{2}$ can be selectively employed depending on workload requirements: the extended analog conductance window enables stable inference for complex tasks with substantially reduced inference power, while current‐path gating through ${\mathrm{Cell}}_{2}$ enables aggressive power reduction for low‐complexity tasks. This dual capability is achieved within a single device structure that remains fully compatible with conventional NOR Flash fabrication flows, requiring no additional masks or process modules. Future work will focus on large‐scale array integration and algorithm–hardware co‐optimization to further extend the task‐adaptive operation enabled by the dual functional roles of ${\mathrm{Cell}}_{2}$.

## 4 Experimental Section

### 4.1 Electrical Measurements

Electrical characterization of the proposed 2M NOR Flash devices was performed using a semiconductor parameter analyzer together with external pulse generators. All measurements were conducted at room temperature under ambient conditions. All measurements, including program, erase, and read operations, were performed on a grounded chuck, so the silicon substrate below the buried oxide (BOX) layer was held at 0 V throughout. Because the active layer is a polycrystalline silicon thin film electrically isolated by the BOX, the device has no separate body terminal. For the transfer characteristics ($I_{D}$–$V_{G}$), the drain voltage was fixed at $V_{D}=1$ V while the gate voltages of the two memory devices were swept simultaneously. For the output characteristics ($I_{D}$–$V_{D}$), the drain voltage was swept while the gate voltages were fixed at $V_{G1}=5$ V for ${\mathrm{Cell}}_{1}$ and $V_{G2}=3.3$ V for ${\mathrm{Cell}}_{2}$. The read current used for conductance‐state definition was defined as the measured drain current at $V_{D}=1$ V under the gate bias conditions $V_{G1}=5$ V and $V_{G2}=3.3$ V. These values were used to construct the conductance lookup table used in the subsequent system‐level simulations. The presented characteristics represent typical device behavior under the described measurement conditions.

### 4.2 Sequential Programming Scheme

Independent programming of the two memory devices in the proposed 2M NOR Flash device was achieved through sequential gate biasing. In the proposed structure, ${\mathrm{Cell}}_{1}$ and ${\mathrm{Cell}}_{2}$ are connected in series between the drain and source terminals, and their gate electrodes are independently accessible through ${\mathrm{Gate}}_{1}$ and ${\mathrm{Gate}}_{2}$, respectively. To establish the conductance states of ${\mathrm{Cell}}_{1}$, programming pulses were applied to ${\mathrm{Gate}}_{1}$ while ${\mathrm{Gate}}_{2}$ was grounded to suppress charge injection into ${\mathrm{Cell}}_{2}$. During this operation, both the drain and source terminals were grounded. The programming pulses had a duration of 100 μs, and the programming voltage applied to ${\mathrm{Gate}}_{1}$ was gradually increased from 5.5 to 10 V in increments of 0.1 V to generate multiple conductance states. After completing the programming of ${\mathrm{Cell}}_{1}$, the conductance states of ${\mathrm{Cell}}_{2}$ were defined while ${\mathrm{Gate}}_{1}$ was grounded to suppress charge injection into ${\mathrm{Cell}}_{1}$. In this step, programming pulses were applied to ${\mathrm{Gate}}_{2}$ while the drain and source terminals were grounded. The same programming scheme was used, where the programming voltage applied to ${\mathrm{Gate}}_{2}$ was increased from 5.5 to 10 V in increments of 0.1 V with a pulse duration of 100 μs.

### 4.3 ${\mathrm{Cell}}_{2}$ Programming for Current‐Path Gating

The programmable states of ${\mathrm{Cell}}_{2}$ were used to implement current‐path gating in the proposed 2M NOR Flash architecture. For pruning experiments, ${\mathrm{Cell}}_{2}$ was programmed into a strongly inhibited state in order to suppress the current path of the device. This state was achieved by applying a programming pulse of 10 V with a duration of 100 μs to ${\mathrm{Gate}}_{2}$. For ${\mathrm{Cell}}_{2}$ state modulation experiments, multiple ${\mathrm{Cell}}_{2}$ conductance states were defined. Eight ${\mathrm{Cell}}_{2}$ states were used in the experiments, consisting of the initial unprogrammed state and seven programmed states generated using programming voltages from 5.5 to 8.5 V with a 0.5 V increment. The initial state was defined as ${\mathrm{Cell}}_{2}$ state 1, while the programmed states were defined as ${\mathrm{Cell}}_{2}$ states 2–8.

### 4.4 Compact Model Construction

A measured‐data‐driven compact current model was constructed using the lookup table data obtained from the proposed devices, with separate models built for the default mode and extended mode current ranges using a common modeling framework. The detailed model construction procedure, including the descriptor extraction, polynomial‐interaction feature basis, and ridge regression with voltage‐dependent and state‐balance weighting, as well as the resulting model accuracy, are provided in the Supporting Information.

### 4.5 Hardware‐Aware Neural Inference

Hardware‐aware neural inference simulations were performed to evaluate the system‐level implications of the proposed device architecture under both functional roles of ${\mathrm{Cell}}_{2}$, namely the analog memory element role and the current‐path gating element role. To evaluate the analog memory element role of ${\mathrm{Cell}}_{2}$, convolutional neural networks including MobileNetV1, VGG‐11, and ResNet‐20 were trained on the CIFAR‐10 dataset. The convolutional backbone served as a feature extractor, and the classification head was implemented as a fully connected layer with non‐negative weights and no bias. During inference, feature vectors were normalized to the voltage range [0, 1] and applied as drain‐input signals. Synaptic weights were mapped to device conductance states, and the resulting drain currents were computed using the compact model. The accumulated currents along bit lines were used to emulate analog multiply–accumulate (MAC) operations. To evaluate the current‐path gating role of ${\mathrm{Cell}}_{2}$, a single‐layer neural network with a 784×10 fully connected structure was trained on the MNIST dataset under the same non‐negative and bias‐free constraints. During inference, input pixels were normalized to [0, 1] and applied as drain‐input voltages, while synaptic weights were mapped to measured conductance states obtained from the device lookup table data.

### 4.6 Power Evaluation

The inference power was estimated using a power proxy defined by the product of the input voltage and the device current. For each synaptic device, the power was calculated using Equation (2):

(2) $$P=V_{\mathrm{in}}\cdot I_{\mathrm{device}},$$

where $V_{\mathrm{in}}$ is the input voltage applied to the drain terminal and $I_{\mathrm{device}}$ is the corresponding drain current. The total power proxy was obtained by summing the contributions from all synaptic devices, as expressed in Equation (3):

(3) $$P_{\mathrm{proxy}}=\sum_{i}V_{\mathrm{in},i}\;I_{\mathrm{device},i}.$$

The reported values correspond to the average over the inference dataset. This metric captures the relative current‐driven power consumption within the analog compute‐in‐memory array and enables consistent comparison across different conductance configurations and ${\mathrm{Cell}}_{2}$ functional roles. It does not include peripheral circuit overhead such as analog‐to‐digital converters and word‐line drivers. A system‐level energy analysis including the word‐line drivers, input and bit‐line drivers, and analog‐to‐digital converters is provided in Section 14 of the Supporting Information.

## Author Contributions

D.R. conceived the idea and fabricated the devices. H.K. assisted with the electrical measurements. S.J. assisted with the TCAD simulations. S.K. performed the experiments, analyzed the data, carried out the system‐level evaluation and modeling, and drafted the manuscript. M.‐H.K. supervised the project and revised the manuscript. All authors discussed the results and approved the final manuscript.

## Conflicts of Interest

The authors declare no conflicts of interest.

## Supporting information


**Supporting File**: advs77797‐sup‐0001‐SuppMat.pdf.

## Data Availability

The data that support the findings of this study are available from the corresponding author upon reasonable request.
