# Supplementary material for: A Dual‐Programmable 2M NOR Flash Architecture for Energy‐Efficient In‐Memory Computing
Source: Adv Sci (Weinh). 2026 Sep 27:e77797. Online ahead of print. doi: 10.1002/advs.77797 (PMC13616378; doi:10.1002/advs.77797)
Supplement: Supplementary file 1 [file ADVS-9999-e77797-s001.pdf]

Supporting Information for
A Dual-Programmable 2M NOR Flash Architecture for Energy-Efficient In-Memory Computing

Contents

1 Device structure and electrical characteristics of the 1M cell 2

2 Fabrication process of the 2M cell 3

3 Two-dimensional current characteristics of the 2M cell 4

4 TCAD simulation of adjacent-cell program/erase disturb 5

5 Retention and endurance of sequentially programmed states 8

6 Device-to-device and cycle-to-cycle variation and usable conductance levels 10

7 Array-level retention, read-disturb, and inter-cell interference characteristics 12

8 Compact model construction and performance evaluation 15
8.1 Compact model construction 15
8.2 Performance evaluation 15

9 Training conditions and hardware-aware inference framework 17
9.1 Model and training parameters 17
9.2 Feature extractor architectures 18
9.3 Hardware-aware inference framework 19

10 Weight representation under the two functional roles of $Cell_2$ 20
10.1 Weight mapping with $Cell_2$ as an analog memory element 20
10.2 Weight mapping with $Cell_2$ as a current-path gating element for pruning and state modulation 20
10.3 Weight distributions and array-level current contribution of near-zero weights . . 23

11 Array-level MAC measurement and linearity analysis 25

12 Variation-aware MAC robustness and Monte-Carlo inference 28
12.1 Post-pruning fine-tuning 30

13 Extension to higher-precision signed operation 33

14 System-level energy analysis including dynamic and peripheral components 34
14.1 Scope and principle 34
14.2 Array static energy 34
14.3 Dynamic charging energy 34
14.4 Peripheral ADC energy 35
14.5 Results 35

15 Benchmark comparison with prior CIM implementations 36

1 Device structure and electrical characteristics of the 1M cell

To examine the physical structure and basic electrical characteristics of the device stack used in this work, structural visualization and transfer-characteristic measurements were performed on a 1M reference cell fabricated with the same stack as that used for the proposed architecture. Figure S1a shows a schematic illustration of the 1M cell used in this study. The figure illustrates the layer configuration of the device stack, including the gate electrode and dielectric layers, as well as the source and drain regions formed on the polycrystalline silicon substrate. Figure S1b presents the transfer characteristics (I_D–V_G) measured at a drain voltage of 1 V after program operations. Program pulses were applied with amplitudes ranging from 6 V to 10.5 V in increments of 0.5 V and a pulse width of 10 ms. As the program-pulse amplitude increases, the transfer curves shift systematically, indicating progressive charge trapping and controllable modulation of the channel conductance. Figure S1c shows the transfer characteristics (I_D–V_G) measured after erase operations. Erase pulses with amplitudes ranging from −6 V to −10.5 V in 0.5 V steps and a pulse width of 10 ms were applied. The erase behavior was evaluated under the same measurement condition of V_D = 1 V, allowing direct comparison with the programmed states. The measured curves confirm that the device conductance can also be modulated in the opposite direction, enabling bidirectional conductance tuning. These results verify that the 1M cell fabricated with the same stack exhibits stable and controllable program/erase characteristics.

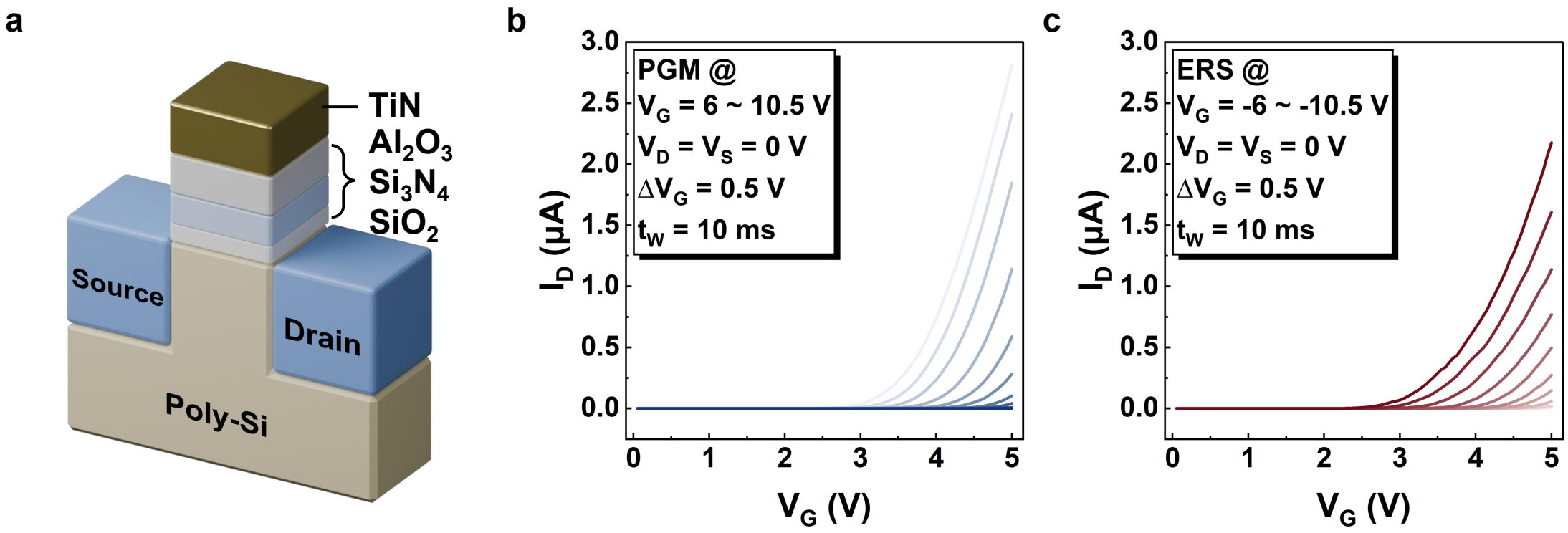

Figure S1. Structure and electrical characteristics of the 1M cell fabricated with the same stack used in this work. (a) Schematic illustration of the 1M cell, showing the layer structure including the gate electrode (TiN), dielectric layers (SiO_2, Si_3N_4, Al_2O_3), and polycrystalline silicon channel with source and drain regions. (b) Transfer characteristics (I_D–V_G) measured at a drain voltage of 1 V for multiple states obtained using program pulses with amplitudes ranging from 6 V to 10.5 V in 0.5 V steps and a pulse width of 10 ms. (c) Transfer characteristics (I_D–V_G) measured at a drain voltage of 1 V for multiple states obtained using erase pulses with amplitudes ranging from −6 V to −10.5 V in 0.5 V steps and a pulse width of 10 ms.

2 Fabrication process of the 2M cell

The fabrication process of the 2M cell used in this work is illustrated in Figure S2. First, a buried oxide (BOX) layer was formed on the silicon substrate to electrically isolate the active region. Next, a polycrystalline silicon (poly-Si) layer was formed to serve as the channel material. The active regions were then defined by patterning and etching processes. After defining the active regions, a gate dielectric stack consisting of SiO_2, Si_3N_4, and Al_2O_3 was deposited to enable charge trapping. Subsequently, a TiN layer was deposited as the gate electrode material. Finally, gate patterning was performed to form two independent gate electrodes corresponding to $Cell_1$ and $Cell_2$.

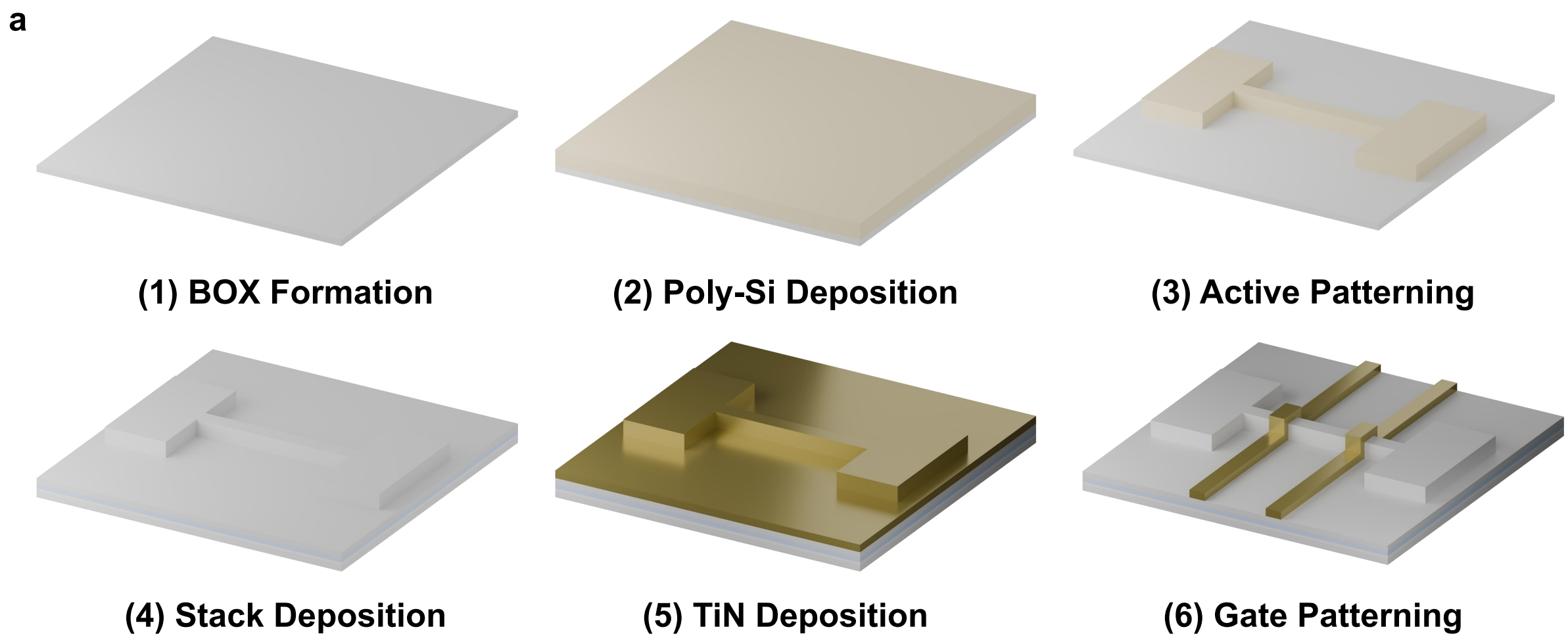

Figure S2. Fabrication process flow of the 2M cell structure. (1) Formation of the buried oxide (BOX) layer. (2) Formation of the polycrystalline silicon channel layer. (3) Definition of the active regions. (4) Deposition of the gate dielectric stack consisting of $SiO_2/Si_3N_4/Al_2O_3$. (5) Deposition of the TiN gate electrode. (6) Gate patterning to form two independent gate electrodes corresponding to $Cell_1$ and $Cell_2$.

3 Two-dimensional current characteristics of the 2M cell

To characterize the dependence of the read current on the programmed states of $Cell_1$ and $Cell_2$, a two-dimensional current mapping was performed by independently programming the two cells. As illustrated in Figure S3a, $Cell_1$ and $Cell_2$ were programmed separately using voltage pulses applied to the corresponding gate terminals while all other terminals were grounded. For both cells, the program voltage was swept from 6 V to 10.5 V in 0.5 V steps, with a pulse width of 10 ms. After programming, the read current was measured under a fixed bias condition. Specifically, the read operation was performed with $V_{\mathrm{G1}} = V_{\mathrm{G2}} = 5$ V and $V_{\mathrm{D}} = 1$ V, while the source was grounded. The resulting read current is shown in Figure S3b as a heatmap with respect to the programmed voltages of $Cell_1$ (V_{P1}) and $Cell_2$ (V_{P2}). The measured current forms a two-dimensional current map, indicating that the overall current is jointly modulated by the states of both cells.

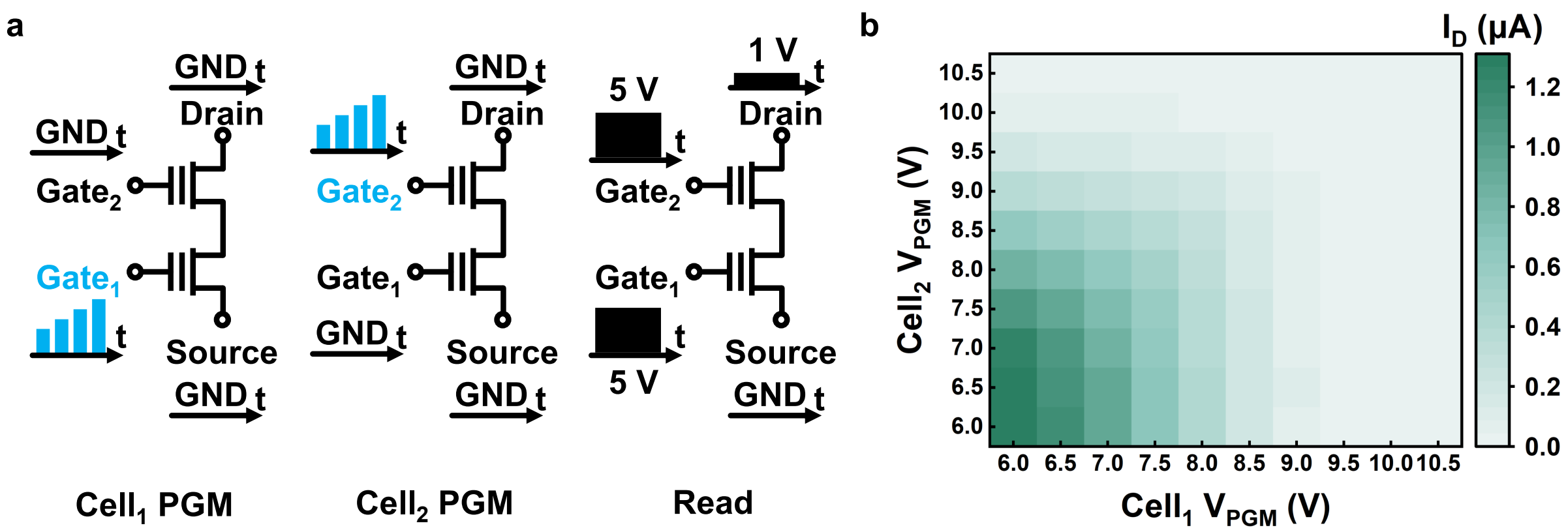

Figure S3. Two-dimensional current characteristics of the 2M cell structure. (a) Measurement scheme for independent programming of $Cell_1$ and $Cell_2$. The program voltage for both cells was swept from 6 V to 10.5 V in 0.5 V steps using 10 ms pulses, while other terminals were grounded. During read operation, $V_{\mathrm{G1}} = V_{\mathrm{G2}} = 5$ V and $V_{\mathrm{D}} = 1$ V were applied. (b) Measured read current (I_D) shown as a heatmap with respect to the programmed voltages of $Cell_1$ (V_{P1}) and $Cell_2$ (V_{P2}). The color scale represents the drain current, illustrating a two-dimensional current map defined by the combined states of the two cells. A coarse voltage step of 0.5 V was used in this measurement to efficiently visualize the two-dimensional current landscape over the full programming range of both cells. Fine-step programming conditions used for the main characterization are described in the Experimental Section of the main text.

4 TCAD simulation of adjacent-cell program/erase disturb

To examine whether the program and erase operations applied to one gate disturb the charge stored under the adjacent gate within the 2M cell, two-dimensional TCAD simulations were performed on the fabricated device structure. Figure S4 shows the simulated structure and the physical parameters used in the simulation. Two independent TiN gates ($Gate_1$ and $Gate_2$) with a gate length of 0.5 µm, separated by a 2 µm gap, share the $SiO_2/Si_3N_4/Al_2O_3$ charge-trap gate stack on a 50 nm polycrystalline silicon channel over a buried oxide, and the trapped charge in the nitride layer under each gate is monitored separately during the program and erase transients. The disturb immunity was examined with the same pulse schemes as the device operation. For the program disturb, both gates first receive a single +5 V, 100 µs pulse to establish the baseline trapped charge, and ISPP pulses from +5.5 to +10 V in 0.5 V steps (100 µs each) are then applied to the aggressor gate only, with the other gate, the source, and the drain grounded. For the erase disturb, both cells are first fully programmed (+10 V, 10 ms), and erase pulses from −5.5 to −10 V in 0.5 V steps (10 ms each) are then applied to the aggressor gate only. After each pulse, the lateral distribution of the trapped electron density in the nitride layer is extracted along a cutline through the nitride spanning both gates. Figure S5 shows the trapped-charge distributions for all four aggressor and victim combinations. During programming (Figure S5a,b), the peak trapped charge under the aggressor gate increases from approximately 1.6×10^8 to 1.7×10^{18} cm^{-3} over the ISPP sweep, by approximately ten orders of magnitude, while the distribution under the adjacent gate remains fixed at its baseline value over the entire sweep. During erase (Figure S5c,d), the peak trapped charge under the aggressor gate is reduced from approximately 2.1×10^{19} to 3.8×10^{14} cm^{-3}, by nearly five orders of magnitude, while the distribution under the adjacent gate changes by less than 0.001%. The distributions also show that the trapped charge remains confined under the respective gates, with no charge accumulation in the 2 µm gap region, and the behavior is identical for the $Gate_1$ and $Gate_2$ aggressor cases, confirming the symmetry of the structure. This immunity follows from the physics of the FN-tunneling-based operation. The tunneling current depends exponentially on the oxide field and is therefore confined to the region directly under the biased gate, whereas the fringing field at the adjacent nitride region, separated by the 2 µm gap, remains far below the FN regime. In addition, the injected charge is stored in the deep traps of the nitride layer and remains spatially localized, without the lateral charge migration of a continuous floating gate. The program and erase operations of one cell therefore do not disturb the charge stored in the adjacent cell, supporting the independent sequential programming of $Cell_1$ and $Cell_2$ employed throughout this work.

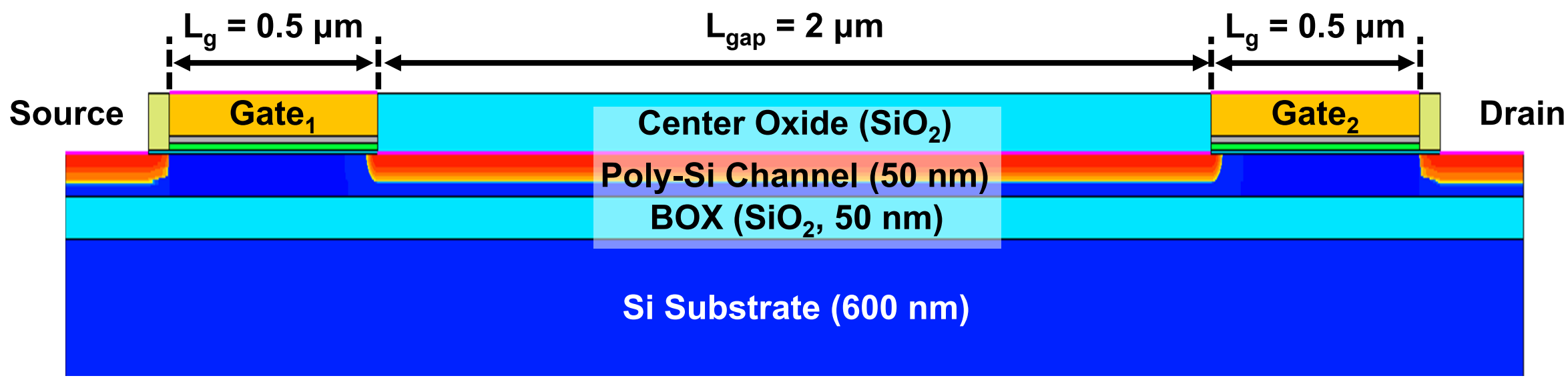

Parameter	Value	Parameter	Value
Gate Length (L_g)	0.5 μm	Tunneling Oxide (SiO_2)	4 nm
Gate Gap (L_{gap})	2.0 μm	Charge Trap (Si_3N_4)	7 nm
Spacer Length (L_{sp})	0.05 μm	Blocking Oxide (Al_2O_3)	9 nm
Poly-Si Thickness	50 nm	TiN Gate Thickness	50 nm
BOX Thickness	50 nm	Channel Doping	8.8×10^{17} cm^{-3}
Si Substrate Thickness	600 nm	S/D Doping	1×10^{20} cm^{-3}
Halo Doping	1.5×10^{18} cm^{-3}	Trap Density	3.0×10^{19} cm^{-3}

Figure S4. Simulated 2M cell structure for the adjacent-cell disturb analysis. Cross-section of the simulated structure with two independent TiN gates ($Gate_1$ and $Gate_2$) on the shared $SiO_2/Si_3N_4/Al_2O_3$ charge-trap stack and the polycrystalline silicon channel, together with the physical parameters used in the simulation.

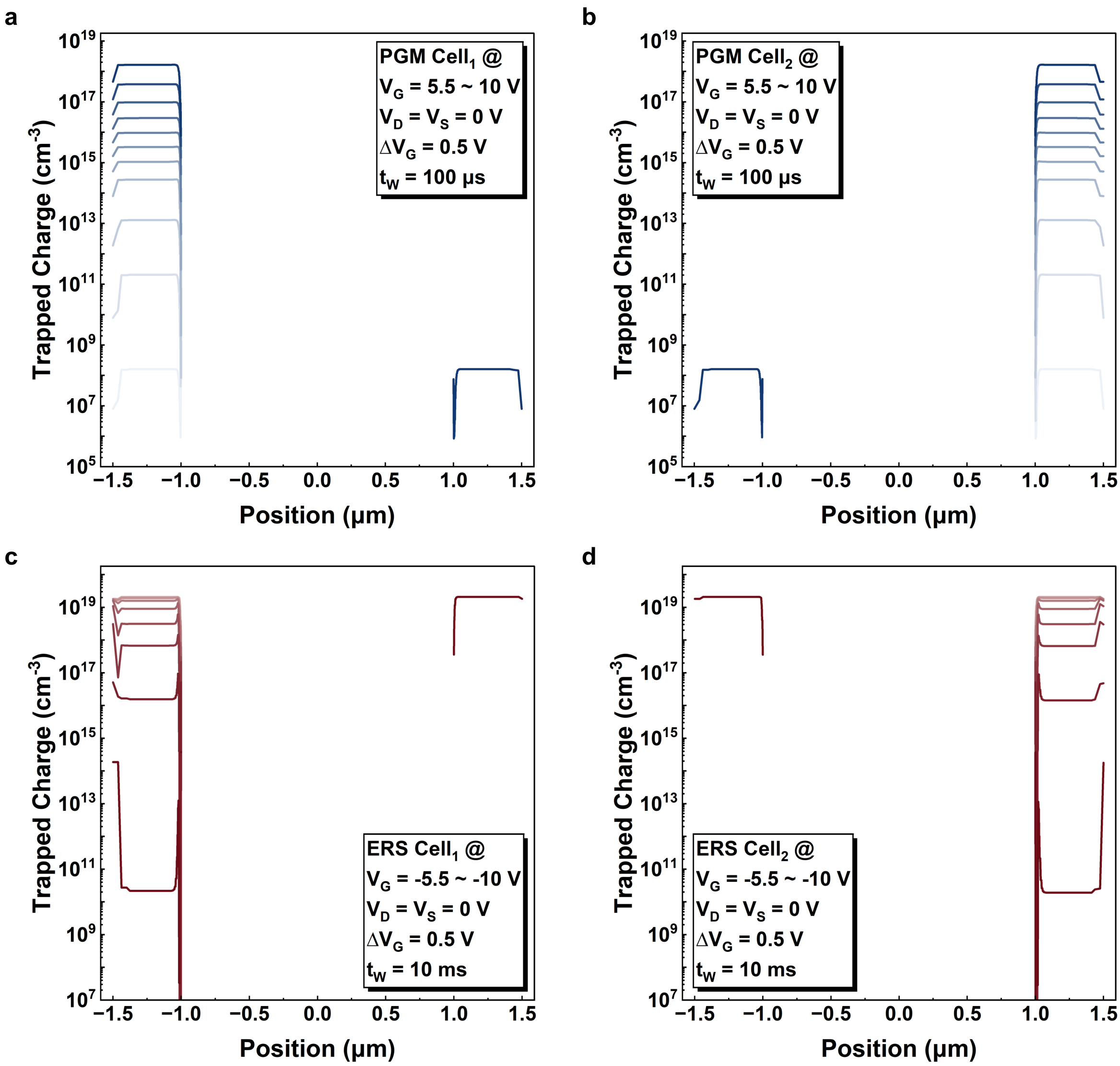

Figure S5. TCAD-simulated adjacent-cell program/erase disturb in the 2M cell. Lateral distributions of the trapped electron density in the Si_3N_4 charge-trap layer, extracted along a cutline through the nitride spanning both gates, after each pulse of the aggressor sequence. (a) $Cell_1$ programming (ISPP, +5.5 to +10 V in 0.5 V steps, 100 µs pulses on $Gate_1$). (b) $Cell_2$ programming (identical scheme applied to $Gate_2$). (c) $Cell_1$ erase (−5.5 to −10 V in 0.5 V steps, 10 ms pulses on $Gate_1$ after both cells are fully programmed). (d) $Cell_2$ erase (identical scheme applied to $Gate_2$). In all four cases, the trapped charge under the pulsed gate is modulated by approximately ten (program) or five (erase) orders of magnitude in peak density, while the distribution under the adjacent gate remains unchanged and no charge accumulates in the gap region.

5 Retention and endurance of sequentially programmed states

To evaluate the long-term stability of the conductance states formed by the sequential programming scheme, retention and endurance measurements were performed on four representative programmed-state combinations, namely S_1 ($Cell_1$ strongly programmed and $Cell_2$ strongly programmed, corresponding to the low-current regime of the extended window), S_2 ($Cell_1$ strongly programmed and $Cell_2$ weakly programmed, an intermediate extended state), S_3 ($Cell_1$ strongly programmed only), and S_4 ($Cell_1$ weakly programmed only). These four states cover both the default states formed by $Cell_1$ alone (S_3 and S_4) and the extended states formed by additional $Cell_2$ programming (S_1 and S_2), allowing a direct evaluation of how sequential programming affects the reliability of the stored states. Figure S6a shows the retention characteristics of the four states measured up to 10^4 s at room temperature under ambient conditions, with $V_{G1} = 5$ V, $V_{G2} = 3.3$ V, and $V_D = 1$ V. All four states remain stable throughout the measurement window, and the ordering and separation of the current levels are preserved. The read-current variations of the $Cell_1$-only states (S_3 and S_4) remain within approximately 1% over 10^4 s, and those of the extended states (S_1 and S_2) remain within approximately 14% and 6%, respectively. These results confirm that sequential programming does not introduce noticeable retention degradation, even in the extended low-current regime. The endurance characteristics of the same four state combinations are shown in Figure S6b, measured up to 10^4 program/erase (P/E) cycles at room temperature under the identical read condition. The read-current variations of the $Cell_1$-only states (S_3 and S_4) remain within approximately 3% over 10^4 cycles, and those of the extended states in which both cells undergo P/E stress (S_1 and S_2) remain within approximately 8%, with the ordering and separation of the current levels preserved throughout the entire cycling window. Because $Cell_1$ and $Cell_2$ share an identical charge-trap gate stack and are operated by the same FN-tunneling-based program/erase scheme, the operating condition in which only $Cell_1$ is programmed and undergoes P/E stress (S_3 and S_4) is electrically equivalent to conventional 1T–1M operation. The absence of additional degradation in the extended states (S_1 and S_2) therefore indicates that the sequential programming of the two cells does not introduce any endurance penalty relative to the conventional configuration. Finally, we note the programming-time cost of the sequential scheme. Reaching the lowest state accessible by $Cell_1$ alone (72.7 nA) requires approximately 4.6 ms per cell under the ISPP conditions described in the Experimental Section of the main text, whereas reaching the lowest state of the extended window (4.22 pA) requires sequential programming of both cells, taking approximately 9.2 ms per cell, corresponding to an approximately 2× programming-time overhead. Because the weights are programmed once when mapped onto the array and only read during inference, this one-time cost does not affect the inference latency or throughput.

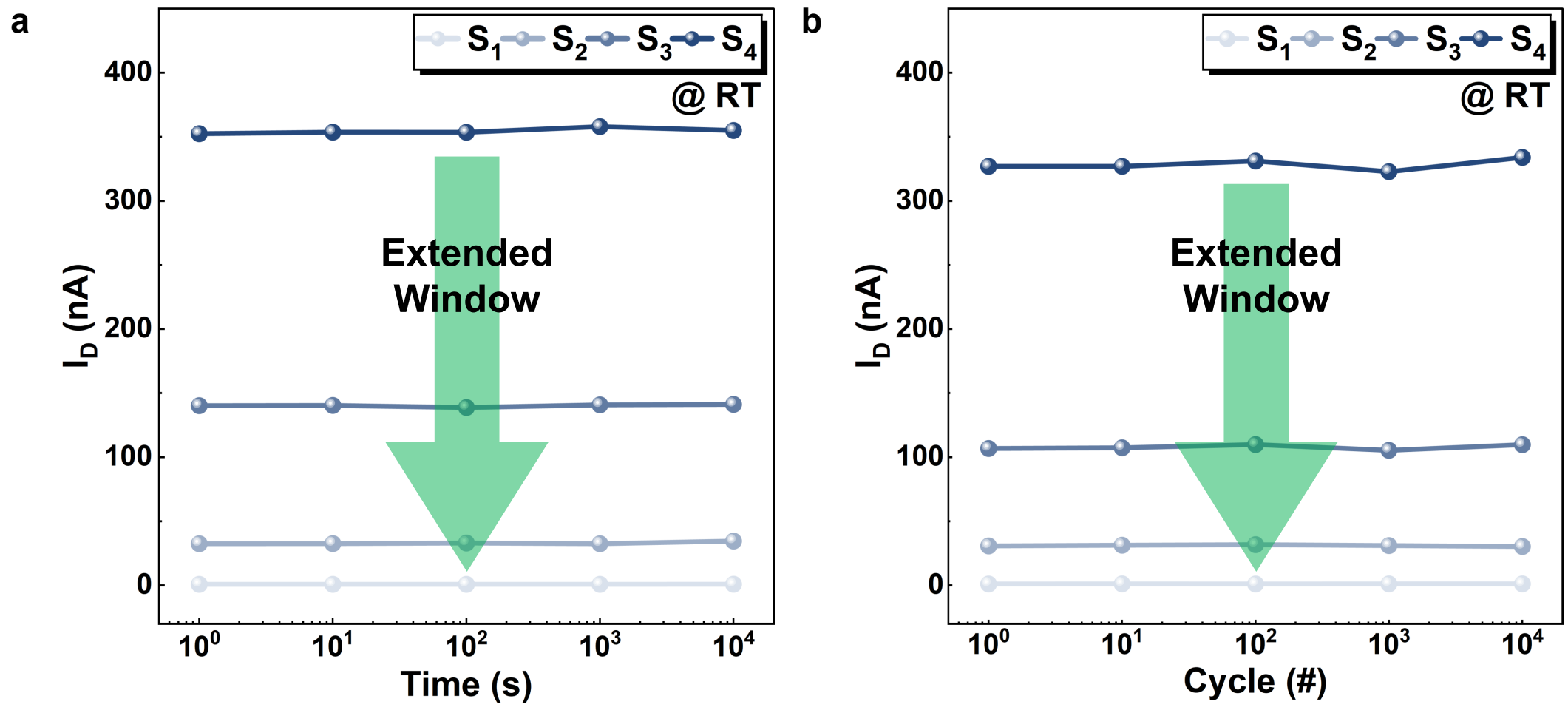

Figure S6. Reliability characteristics of the sequentially programmed states. (a) Retention characteristics of the four representative state combinations S_1 ($Cell_1$ strongly programmed and $Cell_2$ strongly programmed), S_2 ($Cell_1$ strongly programmed and $Cell_2$ weakly programmed), S_3 ($Cell_1$ strongly programmed only), and S_4 ($Cell_1$ weakly programmed only), measured up to 10^4 s at room temperature, with $V_{G1} = 5$ V, $V_{G2} = 3.3$ V, and $V_D = 1$ V. All states retain their programmed current levels over the entire measurement period. (b) Endurance characteristics of the same four state combinations, measured up to 10^4 P/E cycles at room temperature under the identical read condition. No additional degradation is observed in the extended states (S_1, S_2) relative to the $Cell_1$-only states (S_3, S_4).

6 Device-to-device and cycle-to-cycle variation and usable conductance levels

To evaluate the impact of process variation on the conductance-window extension, device-to-device (D2D) and cycle-to-cycle (C2C) variations were measured for four representative states covering both the default and extended regions, namely S_1 ($Cell_1$ strongly programmed and $Cell_2$ strongly programmed), S_2 ($Cell_1$ strongly programmed and $Cell_2$ weakly programmed), S_3 ($Cell_1$ strongly programmed only), and S_4 ($Cell_1$ weakly programmed only). D2D variation was measured over 50 different cells, and C2C variation was measured over 100 program/erase cycles. Figure S7a,b shows the cumulative distributions of the read current for the four states. The measured relative variation (σ/μ) ranges from 2.0–4.6% in the default region to 6.1–9.5% in the extended low-current region. From these measurements, a statistical variation model was constructed as follows. The standard deviation is defined in the $\log_{10}(I)$ domain, corresponding to multiplicative log-normal noise, and the D2D and C2C components are combined by root-sum-of-squares. The σ values are piecewise-linearly interpolated over $\log_{10}(I)$ between the four measured anchor states. Below the lowest measured state (0.8 nA), σ is linearly extrapolated using the slope of the two lowest measured states. We note explicitly that this portion is trend-based rather than measurement-validated, and that the extrapolation increases σ toward lower currents, making it a conservative assumption. This identical variation model is used for all variation-aware analyses in this work. Based on this model, the number of usable conductance levels was quantified. Because each programmed level is not a fixed current value but a statistical distribution broadened by D2D/C2C variation, we define the number of usable levels as the statistical capacity of the window, that is, the maximum number of levels that can be packed into the window such that the spacing between adjacent level means is at least $2 \times (\sigma_i + \sigma_{i+1})$. This margin corresponds to a single-read misclassification probability of approximately 2.3% when a read threshold is placed between adjacent levels. Under this criterion, the default window (72.7 nA–365 nA) supports 13 usable levels, whereas the extended window supports 28 usable levels within the measurement-validated region (down to 0.8 nA), a 2.2× increase, and up to 39 levels for the full window down to 4.22 pA using the trend-extrapolated σ, a 3.0× increase. We note that in every current region, the number of experimentally demonstrated programmed states exceeds this statistical capacity (for example, 19 measured states exist below 0.8 nA, down to 4.2 pA, versus a statistical capacity of 12 levels in that region), confirming that the counted levels correspond to physically accessible states. The increase in usable levels is also robust to the choice of criterion. Tightening the margin to 3σ yields 9 and 26 levels for the default and extended windows, respectively, with the extension ratio remaining approximately 3×. Applying the identical criterion to the accessible current range of each $Cell_2$ state yields 13, 12, 11, 8, 5, 5, 5, and 4 usable levels for $Cell_2$ states 1 through 8, respectively. All of these ranges lie entirely within the measurement-validated current region. As a corollary, for a 32-level equally spaced mapping of the extended window, the floor current (4.22 pA) amounts to only 0.04% of one level spacing (≈11.8 nA), so that even a fluctuation of the floor current by up to three orders of magnitude would remain below a single level spacing. In contrast, the fluctuation of the highest levels, derived from the measured variation at the highest anchor state, reaches a substantial fraction of the spacing. The noise budget of a multi-level mapping is therefore dominated by the high-current levels, which are equally present in the default window, and not by the pA-level floor. To confirm that these statistics transfer to the array environment, the four states were additionally programmed into cells of the fabricated NOR Flash array using the identical 100 µs ISPP pulse scheme and read under the identical condition ($V_{G1} = 5$ V, $V_{G2} = 3.3$ V, $V_D = 1$ V). The D2D statistics were obtained over 30 randomly selected cells per state, and the C2C statistics over 50 P/E cycles, as overlaid on the single-cell distributions

in Figure S7. The measured array-level σ/μ values are 5.2%, 3.8%, 1.5%, and 1.1% (D2D) and 7.9%, 5.3%, 4.8%, and 1.8% (C2C) for S_1 through S_4, respectively, in agreement with the corresponding single-cell values (4.7%, 4.0%, 1.8%, 1.3% and 8.2%, 4.6%, 4.2%, 1.6%) within the sampling uncertainty of the array sample sizes for every state, with array-to-single-cell ratios of 0.80 to 1.15. Recomputing the usable-level count with the array-measured σ values yields 12 levels for the default window and 27 levels for the extended window in the measurement-validated region, preserving the 2.2× increase obtained from the single-cell statistics. Finally, we note the trade-off entailed by the lowered minimum current. As described in Section 5, reaching the lowest state of the extended window requires sequential ISPP programming of both cells, increasing the programming time from approximately 4.6 to 9.2 ms per cell. Because the weights are programmed once when mapped onto the array and only read during inference, this approximately 2× overhead is a one-time cost that does not affect the inference latency or throughput. On the sensing side, the pA-level currents of the deepest extended states demand a larger sensing dynamic range or longer integration when read individually. In the weight mapping of this work, however, these lowest states encode near-zero weights, whose required read precision is the smallest in the array, and, as discussed above, the noise budget of the multi-level mapping is dominated by the high-current levels, which are common to the default window. At the column level, the bit-line current is the sum over all activated cells and remains in the sensing range set by the high-current states, and the analog-to-digital conversion overhead is quantified in Section 14 over a swept range of converter resolutions and efficiencies.

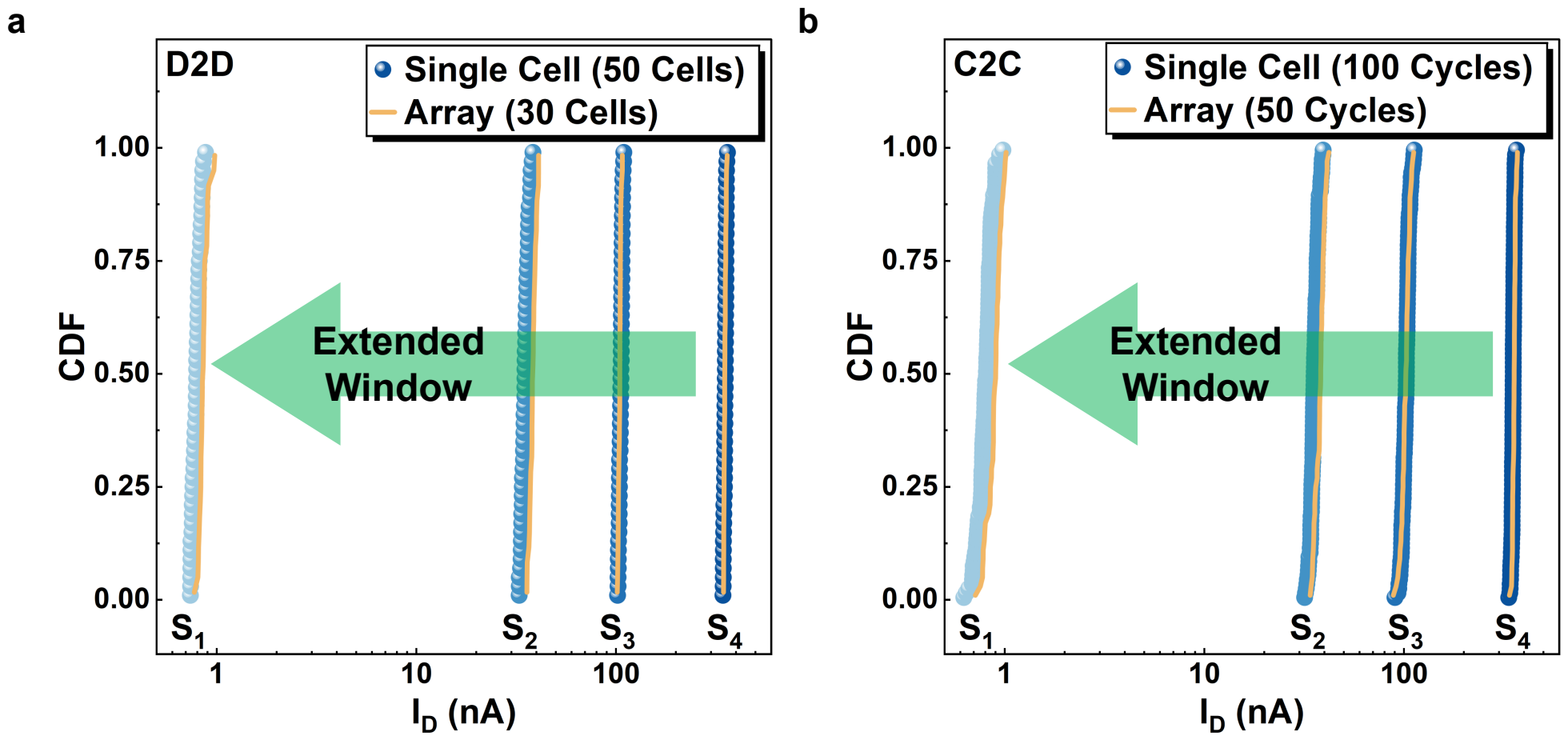

Figure S7. Device-to-device and cycle-to-cycle variation of the programmed states, with the array-level measurements overlaid. (a) Cumulative distributions of the read current over 50 different single cells (D2D, symbols) for the four representative states (S_1–S_4), overlaid with the distributions over 30 randomly selected array cells per state (lines). (b) Cumulative distributions of the read current over 100 program/erase cycles (C2C, symbols) for the same four states, overlaid with the distributions over 50 P/E cycles in the array (lines).

7 Array-level retention, read-disturb, and inter-cell interference characteristics

To further verify that the reliability of the programmed states is preserved in the array environment, retention, read-disturb, and inter-cell interference measurements were performed directly on cells of the fabricated NOR Flash array, using the same four representative state combinations (S_1–S_4) defined in Section 5 and the identical read condition (V_{G1} = 5 V, V_{G2} = 3.3 V, and V_D = 1 V). Figure S8 shows the retention characteristics of the array cells measured up to 10^4 s at room temperature. All four states retain their programmed current levels within approximately 7% over the entire measurement window, with the ordering and separation of the levels preserved, consistent with the single-cell retention characteristics of Section 5. The inter-cell interference during program and erase operations was evaluated directly on the fabricated array, as illustrated in Figure S9a. A target cell and its three nearest neighboring cells are monitored simultaneously. M1 shares the Gate_2 word line and the bit line with the target, M3 shares the Gate_1 word line and the source line, and M2 is the diagonal neighbor sharing no line, so that the gate-stress and line-stress disturb paths of the array operation are all covered. The four array operations (Cell_1 and Cell_2 program and erase) were applied to the target cell with 100 pulses each, under the bias conditions summarized in Figure S9a. As shown in Figure S9b,c, the target cell follows the intended trajectory in every case, with 191 to 82 nA for Cell_1 programming, 159 nA to 38 pA for Cell_2 programming, and full recovery to 206 and 98 nA for the Cell_1 and Cell_2 erase operations, respectively. Meanwhile, the read currents of all neighboring cells remain within approximately 2% of their initial values for every operation, confirming that the program/erase inhibit behavior demonstrated in Figure 6 of the main text is preserved in the actual array environment. The read-disturb characteristics were evaluated in the same array configuration. The identical read operation was repeated 10^3 times on the target cell, and the read currents of the target and the three neighboring cells were compared before and after the repeated reads, for each of the four representative states S_1–S_4 (Figure S9d). All sixteen cell–state combinations, including the extended low-current state S_1, remain within approximately 5% of their initial values, with no systematic direction of the change, confirming that repeated read operations disturb neither the read cell itself nor its neighbors. Consistently, repeating the read operation 10^4 times on programmed array cells changes the read current by less than approximately 5%, with no monotonic drift. This behavior is expected from the operating principle. The read operation uses a drain voltage of at most 1 V with DC gate biases, far below the FN programming regime of 5.5–10 V. The read-to-read dispersion observed across the repeated reads is bounded by the measured C2C variation of Section 6, which therefore already accounts for the read-noise contribution in the usable-level analysis.

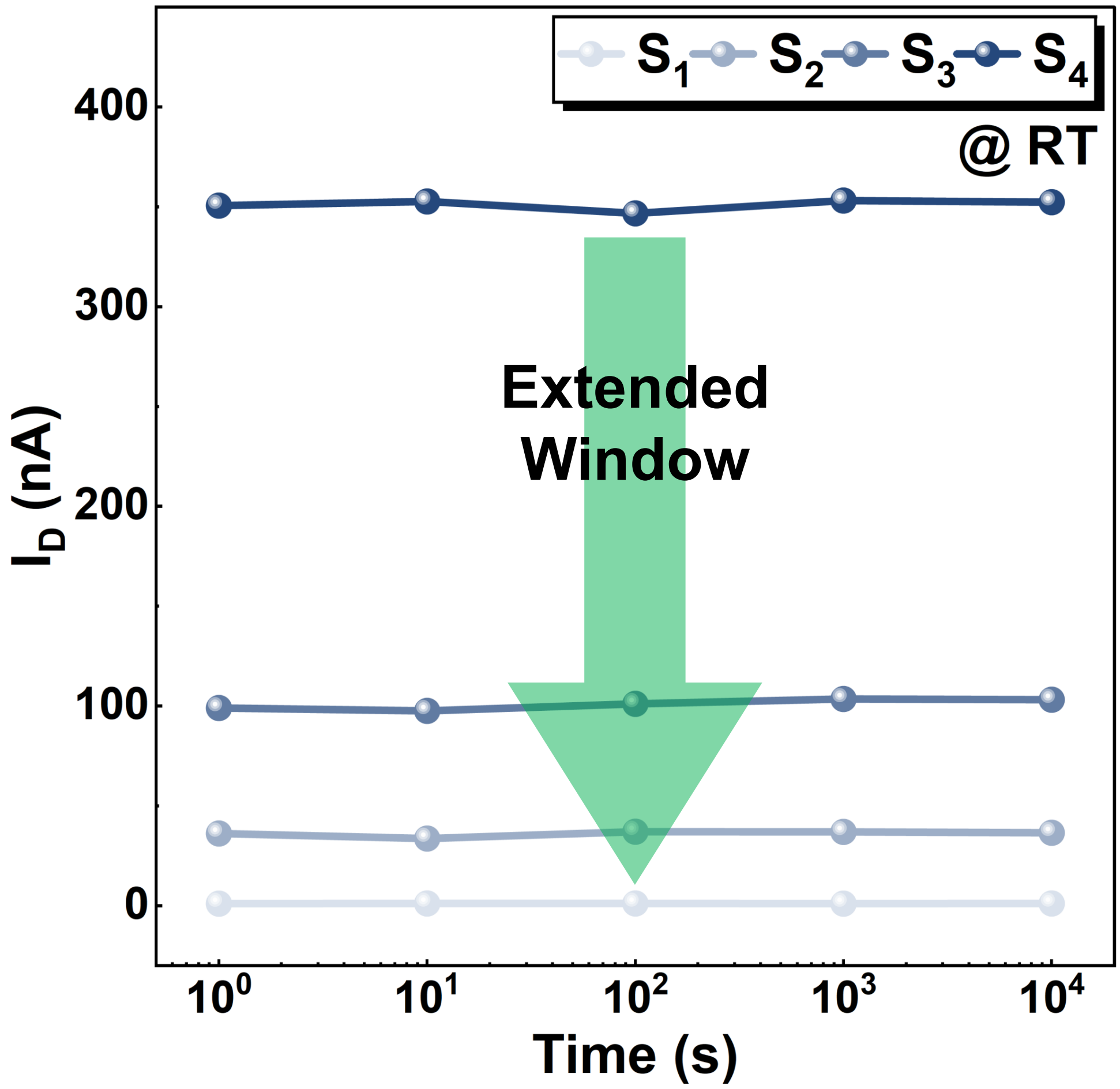

Figure S8. Array-level retention characteristics of the four representative states (S_1–S_4) programmed into cells of the fabricated NOR Flash array, measured up to 10^4 s at room temperature under the identical read condition ($V_{G1} = 5$ V, $V_{G2} = 3.3$ V, and $V_D = 1$ V). All states retain their current levels within approximately 7%.

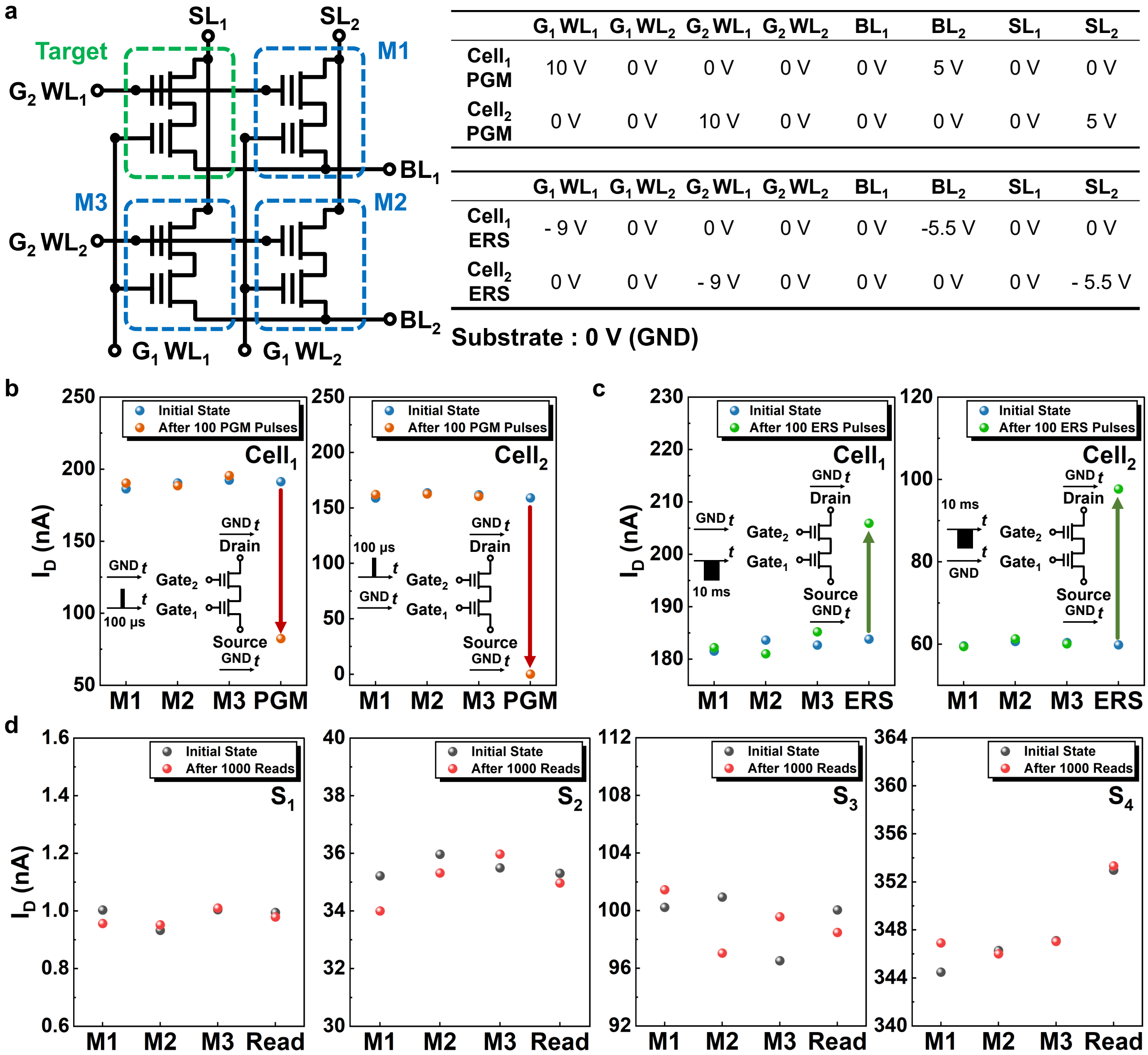

Figure S9. Array-level inter-cell interference and read-disturb characteristics measured on the fabricated NOR Flash array. (a) Array schematic and bias configurations used for the interference evaluation. The target cell and the three neighboring cells are indicated. M1 shares the $Gate_2$ word line and the bit line with the target, M3 shares the $Gate_1$ word line and the source line, and M2 is the diagonal neighbor. The substrate is held at 0 V for all operating modes. (b) Program interference. Read currents of the neighboring cells and the target cell before and after 100 program pulses applied to the target cell, for $Cell_1$ (left) and $Cell_2$ (right) programming. The target cell is programmed as intended (191 to 82 nA for $Cell_1$; 159 nA to 38 pA for $Cell_2$), while all neighboring cells remain within approximately 2% of their initial values. (c) Erase interference. The identical evaluation for the $Cell_1$ (left) and $Cell_2$ (right) erase operations. (d) Read-disturb characteristics. Read currents of the three neighboring cells and the target cell before and after 10^3 repeated read operations, evaluated for the four representative states S_1–S_4. All cell–state combinations remain within approximately 5% of their initial values.

8 Compact model construction and performance evaluation

8.1 Compact model construction

A measured-data-driven compact current model was constructed using the lookup table data obtained from the proposed devices. Separate models were built for the default mode current range and the extended mode current range using their respective datasets while maintaining a common modeling framework. For each conductance state, the measured drain current at $V_D = 1$ V was taken as the anchor current $I_{1\text{V}}$, and each I_D–V_D curve was normalized to a shape function $S(V_D) = I(V_D)/I_{1\text{V}}$, so that the full curve can be reconstructed as $I(V_D) = I_{1\text{V}}S(V_D)$. To capture state-dependent variations in the normalized current shape, four descriptor ratios were extracted from each measured curve:

$$r_k = \frac{I(V_{\text{desc},k})}{I_{1\text{V}}}, \quad k = 1, 2, 3, 4, \tag{1}$$

where $V_{\text{desc},k}$ denotes the k-th descriptor voltage. For the default dataset, all four descriptor voltages were selected from the standard drain-voltage range ($V_{\text{desc},1} < V_{\text{desc},2} < V_{\text{desc},3} < V_{\text{desc},4}$). For the extended dataset, the second descriptor voltage was replaced by a low-voltage point V_{lowextra} positioned below $V_{\text{desc},1}$ to better capture the extended low-current regime. A single global regression model was trained for each dataset to predict the normalized current shape using a polynomial-interaction feature basis constructed from V_D and the four descriptor ratios:

$$\hat{S}(V_D) = \sum_i w_i \, \phi_i(V_D, r_1, r_2, r_3, r_4), \tag{2}$$

where $\{\phi_i\}$ includes polynomial terms up to third order in V_D and pairwise interactions among the descriptor ratios (26 basis terms in total). The regression coefficients $\{w_i\}$ were determined by ridge regression with voltage-dependent weighting to emphasize low-voltage accuracy and state-balance weighting to prevent high-current states from dominating the fit. After training, an anchor correction was applied to enforce $\hat{S}(1\text{ V}) = 1$, and the reconstructed current was obtained as $\hat{I}(V_D) = I_{1\text{V}}\hat{S}(V_D)$.

8.2 Performance evaluation

The accuracy of the compact model was evaluated by comparing reconstructed current characteristics with measured lookup table data across all conductance states. The evaluation was conducted for both the default conductance range and the extended range obtained after additional programming of Cell_2. For each conductance state, the reconstructed I_D–V_D curve was compared with the corresponding measured curve over the full drain-voltage range. To analyze model accuracy under different operating conditions, the error was evaluated separately in two voltage regions. The low-voltage region was defined as $V_D \leq 0.10$ V, while the high-voltage region was defined as $V_D \geq 0.20$ V. Model accuracy was quantified using multiple complementary metrics in the linear current domain, including the root-mean-square error (RMSE), the mean absolute percentage error (MAPE), and the coefficient of determination (R^2). Because the current magnitude becomes extremely small in the low-voltage regime, care was taken to suppress artificial amplification of relative error by applying a minimum current threshold when evaluating the MAPE. To evaluate the agreement of the normalized current profiles independently of the absolute current magnitude, a shape-error metric was defined as the integral of the absolute difference between the measured and reconstructed normalized curves across the drain-voltage range. Multiple descriptor-voltage combinations were scanned to determine the optimal descriptor configuration for both datasets. Candidate configurations were ranked us-

ing a combined score reflecting the full-range error, high-voltage error, low-voltage error, and normalized RMSE. The descriptor configuration with the lowest score was selected as the final compact-model parameter set. Using this optimized configuration, the reconstructed current curves exhibited excellent agreement with the measured lookup table data. For the default dataset, the coefficient of determination reached $R^2 = 0.9996$ over the full voltage range and $R^2 = 0.9991$ in the high-voltage region. The mean absolute percentage error in the high-voltage region was approximately 0.68%, while the normalized shape error was 4.18×10^{-3}. For the extended dataset, which includes the extended low-current regime enabled by Cell_2 programming, the compact model also showed strong agreement with the measured data. The full-range coefficient of determination was $R^2 = 0.996$, and the high-voltage region showed $R^2 = 0.989$. The mean absolute percentage error in the high-voltage region was 1.86%, while the normalized shape error was 1.29×10^{-2}. These results confirm that the compact model accurately reproduces both the magnitude and the voltage-dependent shape of the measured device current across the entire conductance range. The validated compact model was therefore used for the hardware-aware inference analysis presented in the main text. Table S1 summarizes the compact model accuracy metrics for the default and extended conductance ranges.

Table S1. Compact model accuracy metrics for the default and extended conductance ranges.

**Metric**	**Default**	**Extended**
R^2 (full voltage range)	0.9996	0.996
R^2 (high-voltage region)	0.9991	0.989
MAPE (high-voltage region)	0.68%	1.86%
Normalized shape error	4.18×10^{-3}	1.29×10^{-2}

9 Training conditions and hardware-aware inference framework

9.1 Model and training parameters

The CIFAR-10 convolutional neural networks were trained using the Adam optimizer with an initial learning rate of 1×10^{-3} and a batch size of 128 for a maximum of 120 epochs. Early stopping based on the validation accuracy with a patience of 20 epochs was employed to prevent overfitting. During training, horizontal flipping and random translation were used for data augmentation. Separately, the learning rate was reduced by a factor of 0.5 when the validation loss plateaued for 8 consecutive epochs. A validation split of 0.1 and a weight decay of 5×10^{-4} were used during training. All dense layers employed a non-negative kernel constraint so that the trained weights could be directly mapped to physically accessible conductance states of the memory cells. Bias terms were disabled, and the output layer produced logits without applying a softmax activation. The evaluated models consisted of MobileNetV1, a VGG-11-style network, and ResNet-20, each followed by global average pooling and a Dense(10) classifier. For the MNIST experiments, a single fully connected model consisting of a 784-to-10 linear layer with non-negative weights and no bias term was used. The model was trained using the Adam optimizer with a learning rate of 1×10^{-3}, a batch size of 256, and 200 training epochs. Input images were normalized to the range $[0, 1]$ and quantized to a 51-level voltage grid with a step size of 0.02 V to match the voltage grid used in the measured device lookup table during hardware-aware inference. Table S2 summarizes the CIFAR-10 convolutional neural network architectures used for hardware-aware inference evaluation. Table S3 summarizes the common training hyperparameters used for CIFAR-10 model training.

Table S2. CIFAR-10 convolutional neural network architectures used for hardware-aware inference evaluation.

Backbone / head	Bias	Total params	Trainable params
MobileNetV1 ($\alpha = 0.5$), GAP $\rightarrow$ Dense(10)	Off	834,656	823,712
VGG-11-style backbone, GAP $\rightarrow$ Dense(10)	Off	9,233,856	9,228,352
ResNet-20 backbone, GAP $\rightarrow$ Dense(10)	Off	274,032	272,464

Table S3. Common training hyperparameters used for CIFAR-10 model training.

Item	Value
Optimizer	Adam
Learning rate	1×10^{-3}
Batch size	128
Maximum epochs	120
Weight decay	5×10^{-4}
Validation split	0.1
Data augmentation	Horizontal flip + translation
Output type	Logits (no softmax)
Dense constraint	Non-negative kernel constraint

9.2 Feature extractor architectures

The detailed architectures of the feature extractors are summarized in Tables S4–S6.

Table S4. Feature extractor architecture of MobileNetV1 used for CIFAR-10.

**Stage**	**Operation**	**Stride**	**Output size**
Input	Image	–	$32 \times 32 \times 3$
1	Conv 3×3	2	$16 \times 16 \times 16$
2	Depthwise separable convolution	1	$16 \times 16 \times 32$
3	Depthwise separable convolution	2	$8 \times 8 \times 64$
4	Depthwise separable convolution	1	$8 \times 8 \times 64$
5	Depthwise separable convolution	2	$4 \times 4 \times 128$
6	Depthwise separable convolution $\times 4$	1	$4 \times 4 \times 128$
7	Depthwise separable convolution	2	$2 \times 2 \times 256$
8	Depthwise separable convolution	1	$2 \times 2 \times 256$
9	Depthwise separable convolution	2	$1 \times 1 \times 512$
10	Global average pooling	–	512

Table S5. Feature extractor architecture of VGG-11-style network used for CIFAR-10.

**Stage**	**Operation**	**Stride**	**Output size**
Input	Image	–	$32 \times 32 \times 3$
1	Conv 3×3, BN, ReLU	1	$32 \times 32 \times 64$
1	Max pooling	2	$16 \times 16 \times 64$
2	Conv 3×3, BN, ReLU	1	$16 \times 16 \times 128$
2	Max pooling	2	$8 \times 8 \times 128$
3	Conv 3×3, BN, ReLU $\times 2$	1	$8 \times 8 \times 256$
3	Max pooling	2	$4 \times 4 \times 256$
4	Conv 3×3, BN, ReLU $\times 2$	1	$4 \times 4 \times 512$
4	Max pooling	2	$2 \times 2 \times 512$
5	Conv 3×3, BN, ReLU $\times 2$	1	$2 \times 2 \times 512$
6	Global average pooling	–	512

Table S6. Feature extractor architecture of ResNet-20 used for CIFAR-10.

**Stage**	**Operation**	**Stride**	**Output size**
Input	Image	–	$32 \times 32 \times 3$
1	Conv 3×3	1	$32 \times 32 \times 16$
2	Residual block (3 blocks)	1	$32 \times 32 \times 16$
3	Residual block (3 blocks)	2	$16 \times 16 \times 32$
4	Residual block (3 blocks)	2	$8 \times 8 \times 64$
5	Global average pooling	–	64

9.3 Hardware-aware inference framework

For hardware-aware inference, the trained software weights were mapped to discrete memory-cell states using either experimentally measured lookup table data or the validated compact model. For the CIFAR-10 experiments, the trained dense-layer weights were mapped to the available memory states. Input activations were encoded as drain voltages, and the corresponding drain currents were obtained using the compact model. The output class scores were computed by accumulating the resulting currents for each output node. For the MNIST experiments, inference was implemented by directly using lookup table data obtained from device measurements. Input pixels were quantized to a 51-level drain-voltage grid with a step size of 0.02 V, consistent with the device measurement conditions. The trained 784-to-10 non-negative weight matrix was mapped to programmable cell states using rank-based quantization. During inference, the current corresponding to each input voltage and mapped state was retrieved from the lookup table and accumulated to obtain the output scores. Figure S10 shows the training curves of the neural network models used in this work.

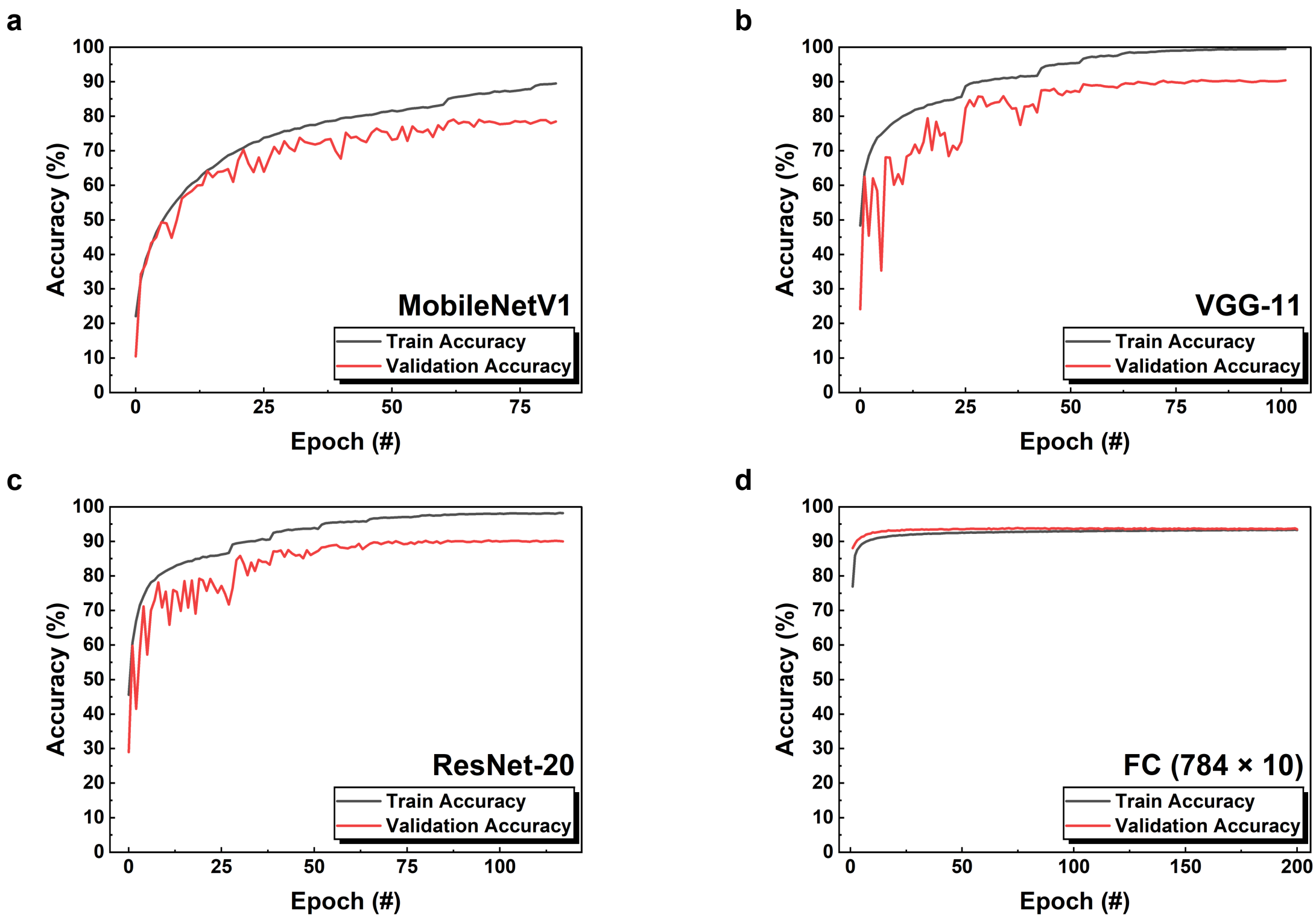

Figure S10. Training curves of the neural network models used in this work. (a) MobileNetV1 trained on CIFAR-10. (b) VGG-11-style convolutional network trained on CIFAR-10. (c) ResNet-20 trained on CIFAR-10. (d) Fully connected 784×10 model trained on MNIST. Black and red curves indicate training and validation accuracy, respectively.

10 Weight representation under the two functional roles of $Cell_2$

To analyze how trained weights are represented under the two functional roles of $Cell_2$, weight distributions were visualized as heatmaps at various stages of the mapping process. For each neural network model, three representations were considered, namely the original floating-point weights, the quantized weights, and the hardware-mapped weights corresponding to physical conductance values. The mapping was performed using the measured conductance space of the 2M cell. In the hardware-mapped weight heatmaps, the color scale represents the device conductance in units of nanosiemens (nS), defined as $G = I_{\mathrm{D}}/V_{\mathrm{D}}$ at $V_{\mathrm{D}} = 1$ V.

10.1 Weight mapping with $Cell_2$ as an analog memory element

Figure S11 shows the weight distributions for three representative models, MobileNetV1, VGG-11, and ResNet-20, when $Cell_2$ is used as an analog memory element. For each model, the first column represents the original weights, the second column shows the quantized weights, and the third column shows the hardware-mapped weights. The hardware-mapped weights are further divided into two cases, the default conductance range and the extended conductance range enabled by additional programming of $Cell_2$. The extended conductance range reflects the conductance-window extension enabled by utilizing $Cell_2$. As observed in the heatmaps, the extended range enables a broader distribution of conductance values, allowing better representation of the original weight distribution. This result indicates that memory-window extension improves the dynamic range of the mapped weights and reduces quantization-induced distortion.

10.2 Weight mapping with $Cell_2$ as a current-path gating element for pruning and state modulation

To further analyze the behavior of the proposed architecture when $Cell_2$ is used as a current-path gating element, weight distributions were visualized for a fully connected neural network used for MNIST inference. Figure S12a shows the original floating-point weight distribution of the trained 784×10 network. Figure S12b shows the corresponding hardware-mapped weights obtained using the measured conductance space of the 2M cell. To investigate the effect of pruning strategies, Figure S12c compares two pruning approaches with a pruning ratio of 60%, namely minimum-conductance-state-based pruning and $Cell_2$ OFF-state-based true-off pruning. Figure S12d and S12e show the effect of $Cell_2$ state modulation on the mapped weights, comparing $Cell_2$ State 1 and State 8 for 3-bit and 4-bit quantization. The results indicate that the state of $Cell_2$ significantly influences the effective dynamic range of the mapped weights.

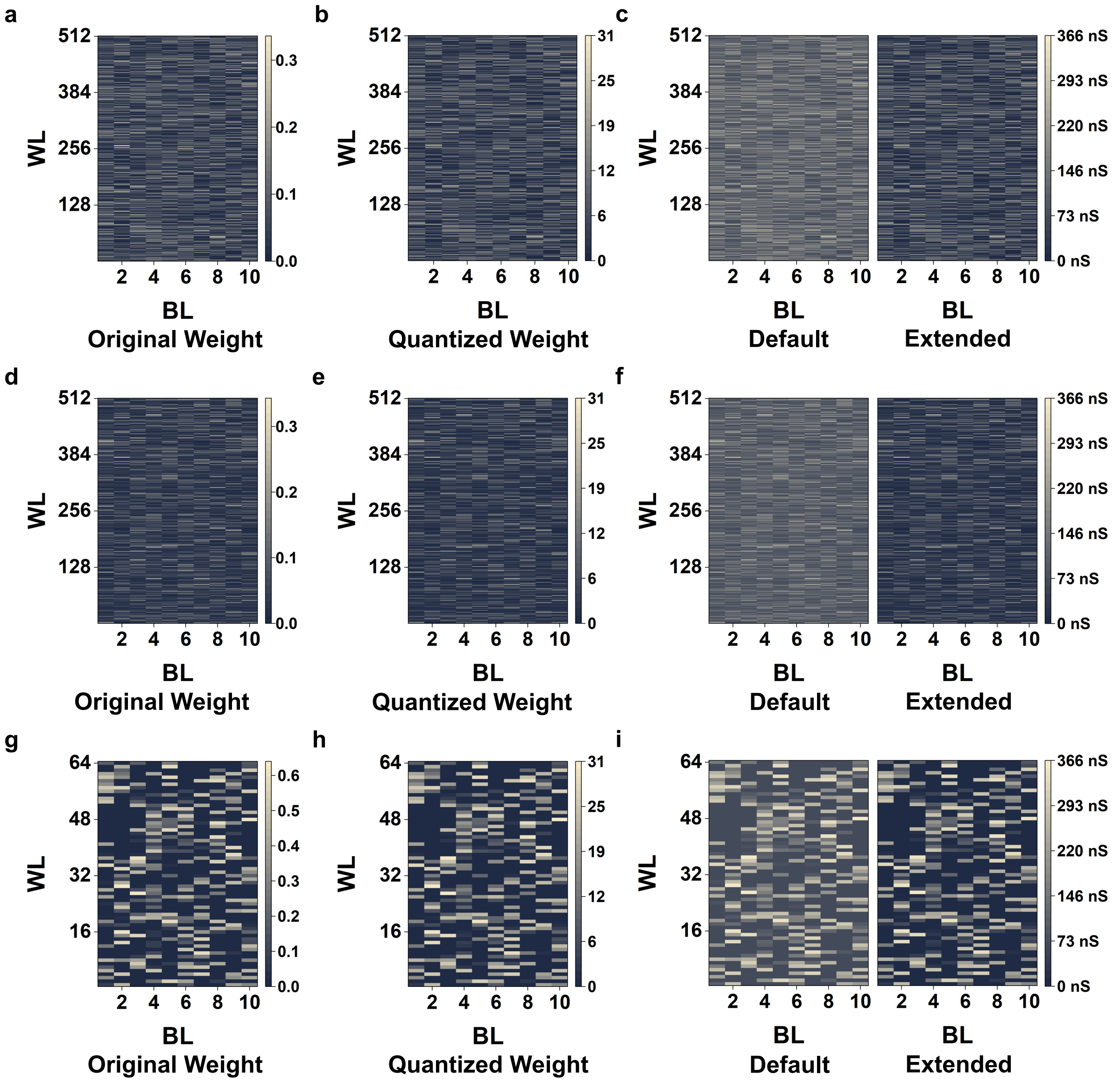

Figure S11. Weight mapping visualization with $Cell_2$ used as an analog memory element. (a–c) MobileNetV1, (d–f) VGG-11, and (g–i) ResNet-20. For each model, the first column shows the original floating-point weights, the second column shows the quantized weights, and the third column shows the hardware-mapped weights. In the hardware-mapped weights, the left panel corresponds to the default conductance range, and the right panel corresponds to the extended conductance range enabled by the use of $Cell_2$. The extended range demonstrates a wider conductance distribution, reflecting improved representation of the original weights.

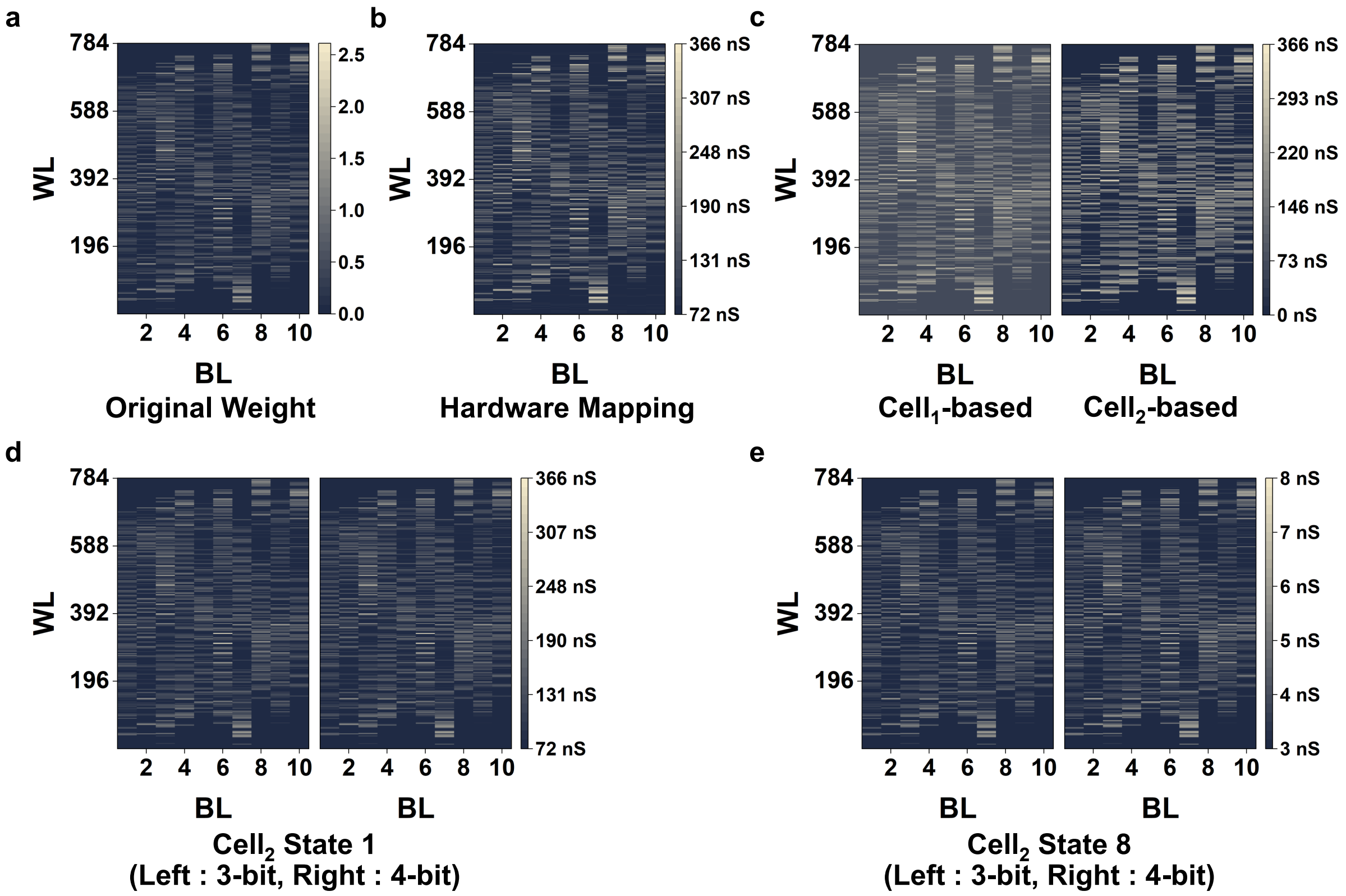

Figure S12. Weight mapping analysis for the MNIST 784 × 10 neural network with Cell_2 used as a current-path gating element. (a) Original floating-point weight distribution. (b) Hardware-mapped weight distribution using the measured conductance space of the 2M cell. (c) Comparison of pruning strategies with a pruning ratio of 60%, showing minimum-conductance-state-based pruning (left) and Cell_2 OFF-state-based true-off pruning (right). (d) Hardware-mapped weights with Cell_2 fixed at State 1 for 3-bit (left) and 4-bit (right) quantization. (e) Hardware-mapped weights with Cell_2 fixed at State 8 for 3-bit (left) and 4-bit (right) quantization.

10.3 Weight distributions and array-level current contribution of near-zero weights

To quantify the array-level impact of the residual current of cells mapped to near-zero weights, the weight distributions of the trained networks were analyzed. Figure S13a shows the distributions of the trained dense-layer weights for the four non-negative-constrained networks used in this work (MobileNetV1, VGG-11, ResNet-20, and the 784×10 MNIST classifier), together with a conventionally trained (unconstrained) 784×10 MNIST network for comparison. In all cases, the majority of the weights are concentrated near zero. For the non-negative networks, 47–68% of the weights fall within $|w| < 0.05$ of the weight range, and the unconstrained network likewise exhibits a distribution sharply peaked at zero. Because analog in-memory computing reads all cells simultaneously, the cells mapped to these near-zero weights constitute a large fraction of the array. The consequence for array-level power is quantified in Figure S13b. Mapping the weights linearly onto the conductance range, cells with normalized weights below 0.05 account for 34–59% of all cells. Under the conventional single-cell mapping, in which the minimum read current is limited to 72.7 nA, these near-zero cells consume 22–41% of the total array current despite carrying almost no information. With the extended window enabled by the proposed architecture (minimum read current of 4.22 pA), the current share of the same cells drops to 0.5–3.4%. The resulting first-order estimate of the total current reduction (approximately 45–66%) is consistent with the hardware-aware inference results reported in the main text (41.7–50.2%, Figure 7c), independently confirming that the suppression of the near-zero-weight current is the dominant origin of the power benefit.

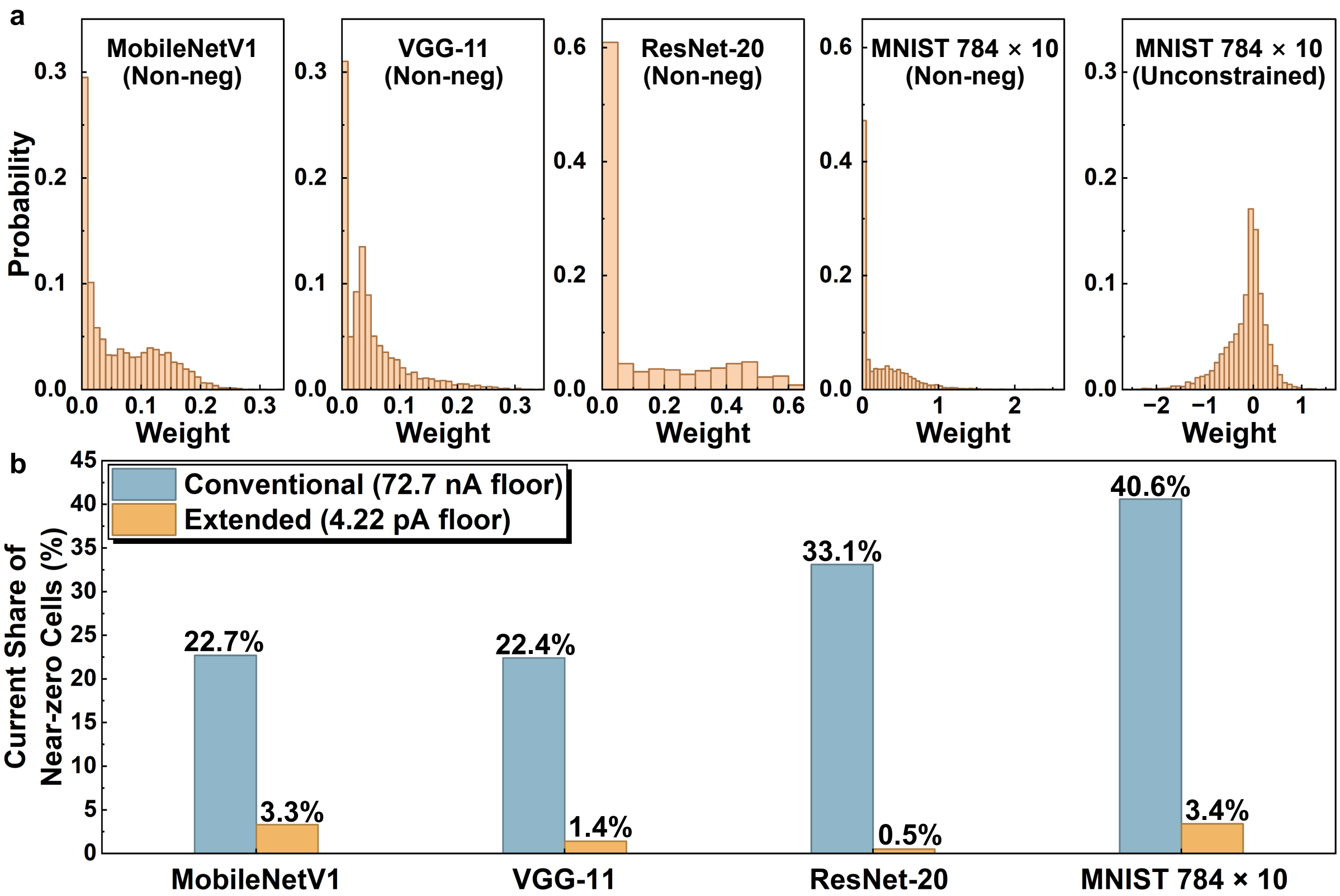

Figure S13. Weight distributions and array-level current contribution of near-zero weights. (a) Distributions of the trained dense-layer weights for the non-negative-constrained networks used in this work (MobileNetV1, VGG-11, ResNet-20, and the 784×10 MNIST classifier) and for a conventionally trained (unconstrained) 784×10 MNIST network. All distributions are concentrated near zero. (b) Fraction of the total array current consumed by near-zero-weight cells (normalized weight < 0.05) under the conventional single-cell mapping (minimum current 72.7 nA) and under the extended window (4.22 pA), for each network.

11 Array-level MAC measurement and linearity analysis

To verify the validity of the drain-input encoding scheme under the nonlinear I_D–V_D characteristics of the device, the multiply–accumulate (MAC) operation was evaluated at two complementary levels, by direct array-level measurement and by simulation against a perfectly linear MAC. The two analyses isolate different non-idealities, as described below. Figure S14 shows the MAC operation measured directly on the fabricated array, in which different input voltages were applied to different rows and the summed current was measured on the shared source-side line. Here, the ideal values are computed from the per-cell lookup-table currents at the applied inputs, so the device I–V characteristics are already included in the reference. The measured deviation therefore directly quantifies how much the array-level effects, such as line resistance, degrade the ideality of the MAC operation. The measured MAC outputs exhibit a correlation of $R^2 = 0.996$ with respect to the ideal values over 8,080 measured points, with an error distribution of $\mu = -1.21\%$ and $\sigma = 0.93\%$ of full scale, experimentally confirming that both the current summation and the linearity of the MAC operation are valid in the actual array environment and that the array-level effects are well controlled. The impact of the device nonlinearity itself was isolated by simulation. Using the measured lookup table, the nonlinear MAC was compared with a perfectly linear MAC under identical conditions over the full MNIST test set (10,000 images). Each MAC output is normalized to its own full-scale maximum, $Y_{\mathrm{norm}} = Y/Y_{\mathrm{max}}$, so that the shape difference between the two MAC outputs is directly compared. The error is defined as the difference of the normalized outputs in percent of full scale. The nonlinear MAC outputs correlate with the ideal linear MAC with $R^2 = 0.962$ (Figure S16a), and the classification accuracy of 92.30% is nearly identical to that of the ideal linear case (92.23%), confirming that the device nonlinearity does not degrade inference accuracy. We note that the two analyses show deviations of opposite signs. The array-level measurement shows a small negative mean error ($\mu = -1.21\%$), whereas the nonlinear MAC in the simulation shows a small positive bias relative to the ideal linear MAC. This is expected rather than contradictory, because the two analyses isolate different non-idealities. The concave (saturation-induced) curvature of the device I–V produces the positive bias relative to a perfectly linear MAC, whereas the array-level parasitics produce the negative bias relative to the lookup-table-based reference. The small magnitude of both deviations confirms that each non-ideality is individually well controlled. In addition, the linearity can be further enhanced simply by changing the input mapping scheme. Instead of assigning equally spaced input voltages, the input levels are assigned such that the output currents are equally spaced (equal-current input mapping). Since the input encoding is performed by the input DAC, this scheme requires only a different level assignment. The two input-mapping schemes are illustrated schematically in Figure S15. As shown in Figure S16b,c, this approach improves the correlation with the ideal linear MAC from $R^2 = 0.962$ to $R^2 = 0.997$ and reduces the MAC error standard deviation from 0.79% to 0.26% of full scale.

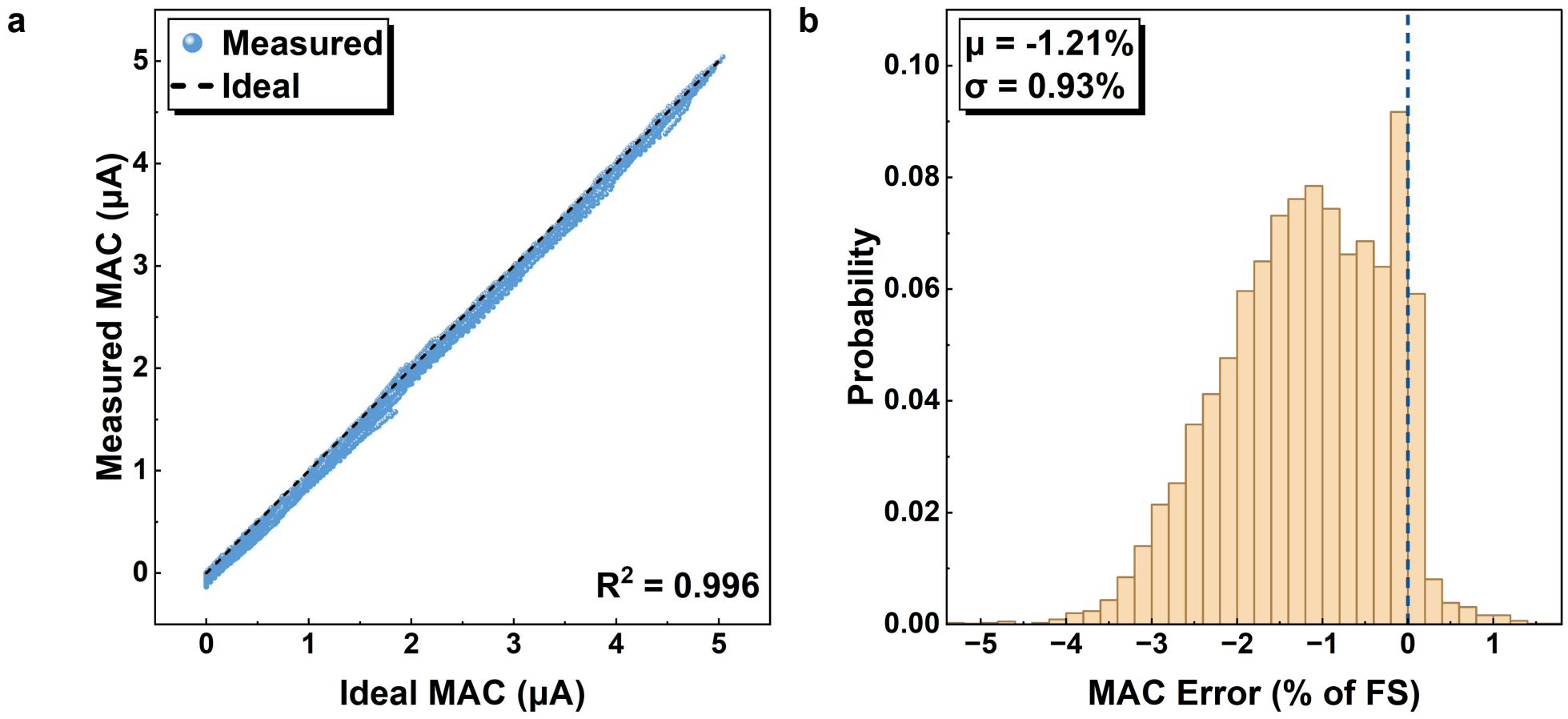

Figure S14. Array-level MAC measurement. (a) Measured MAC outputs versus ideal MAC values over 8,080 measured points, showing a correlation of $R^2 = 0.996$. The dashed line indicates the ideal relationship. The ideal values are computed from the per-cell lookup-table currents at the applied inputs, so the deviation from the dashed line reflects array-level effects such as line resistance. (b) Distribution of the MAC error in percent of full scale, with $\mu = -1.21\%$ and $\sigma = 0.93\%$.

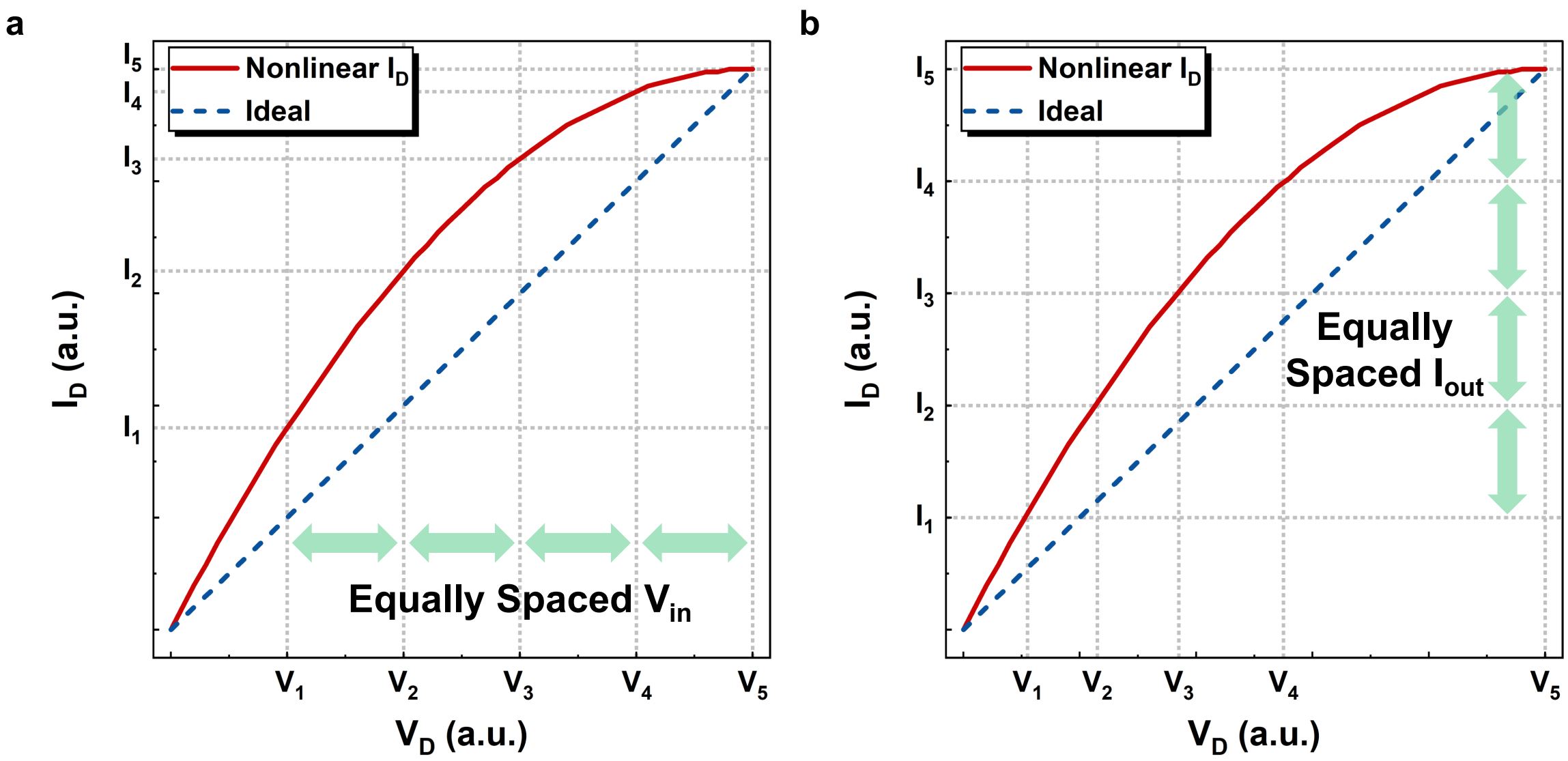

Figure S15. Schematic illustration of the two input-mapping schemes on the nonlinear I_D–V_D characteristic. (a) Equally spaced input voltages. Uniformly spaced input levels (V_1–V_5) produce non-uniformly spaced output currents (I_1–I_5) owing to the concave I_D–V_D curvature. (b) Equal-current input mapping. The input-voltage levels are assigned such that the output currents are equally spaced, compensating the device nonlinearity by construction. The dashed line indicates the ideal linear characteristic.

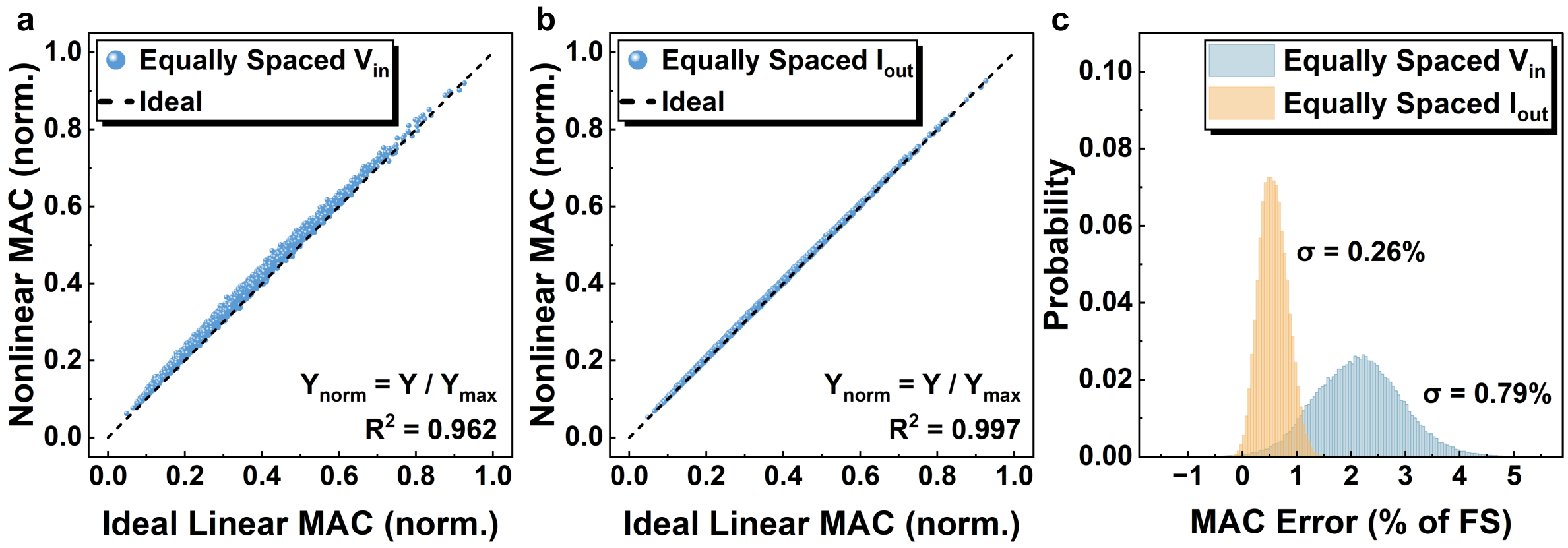

Figure S16. Linearity analysis of the nonlinear MAC based on the measured lookup table. (a) Simulated nonlinear MAC outputs versus ideal linear MAC outputs with equally spaced input voltages ($R^2 = 0.962$). (b) Simulated nonlinear MAC outputs with equal-current input mapping, in which the input levels are assigned such that the output currents are equally spaced ($R^2 = 0.997$). (c) Distributions of the MAC error for the two input-mapping schemes, showing that the equal-current mapping reduces the error standard deviation from 0.79% to 0.26% of full scale. Each MAC output is normalized to its own full-scale maximum ($Y_{\mathrm{norm}} = Y/Y_{\mathrm{max}}$), and the ideal reference is a perfectly linear MAC under identical conditions.

12 Variation-aware MAC robustness and Monte-Carlo inference

To evaluate the impact of the measured variation on array-level computation, the variation model constructed from the D2D/C2C measurements (Section 6) was injected into MAC operations and into the hardware-aware inference simulations. The robustness of the MAC operation was first evaluated at the array level. For an identical ideal MAC (fan-in of 256, weight distributions concentrated near zero, inputs uniform in $[0, 1]$), frozen multiplicative log-normal perturbations based on the measured state-dependent σ were applied independently to each cell (30 Monte-Carlo draws, each draw corresponding to one independently realized array instance), using the same non-negative single-cell weight mapping as in the main text. As shown in Figure S17, the extended window reduces the MAC output noise by a factor of 1.59 and improves the MAC SNR by +4.0 dB relative to the default window (21.6 dB to 25.7 dB), consistently across weight mapping precisions of 3–5 bits. The physical origin of this improvement is that although the relative variation (σ/μ) is larger at low currents, the absolute noise current ($\sigma \times I$) is far smaller, and MAC accuracy is governed by the absolute current error relative to the full-scale signal. In the default window, the many cells mapped to near-zero weights sit on the 72.7 nA current floor and inject floor noise that does not cancel. In the extended window, the floor is at the pA level and this contribution is essentially eliminated. Consequently, the window extension makes array-level operation more robust against variation, not less. The same variation model was then incorporated directly into the hardware-aware inference simulations (20 Monte-Carlo trials per condition, using raw hardware outputs without any output-side correction). As shown in Figure S18a, the extended mode maintains classification accuracy equal to or higher than the default mode for all three networks and within 0.6 percentage points of the software baseline (MobileNetV1 78.44 ± 0.07%, VGG-11 89.79 ± 0.03%, and ResNet-20 89.96 ± 0.04%), while reducing the inference power by 43.3–51.5%, consistent with the variation-free results reported in the main text. The two current-path gating functions of $Cell_2$ were also re-evaluated under the measured variation (20 Monte-Carlo trials per condition). As shown in Figure S18b, true-off pruning maintains its power advantage over minimum-conductance-state-based pruning across the entire pruning-ratio range, reducing the inference power to 43% of the unpruned case at a 75% pruning ratio while retaining higher accuracy than the conventional scheme. As shown in Figure S18c, $Cell_2$ state modulation likewise preserves its task-adaptive trade-off. For 4-bit weights, the classification accuracy remains approximately 89–91% up to $Cell_2$ state 7 while the inference power decreases to below 5% of that at state 1. These results confirm that all three functional benefits of the proposed architecture, namely the extended window, true-off pruning, and $Cell_2$ state modulation, remain valid under the measured device variation.

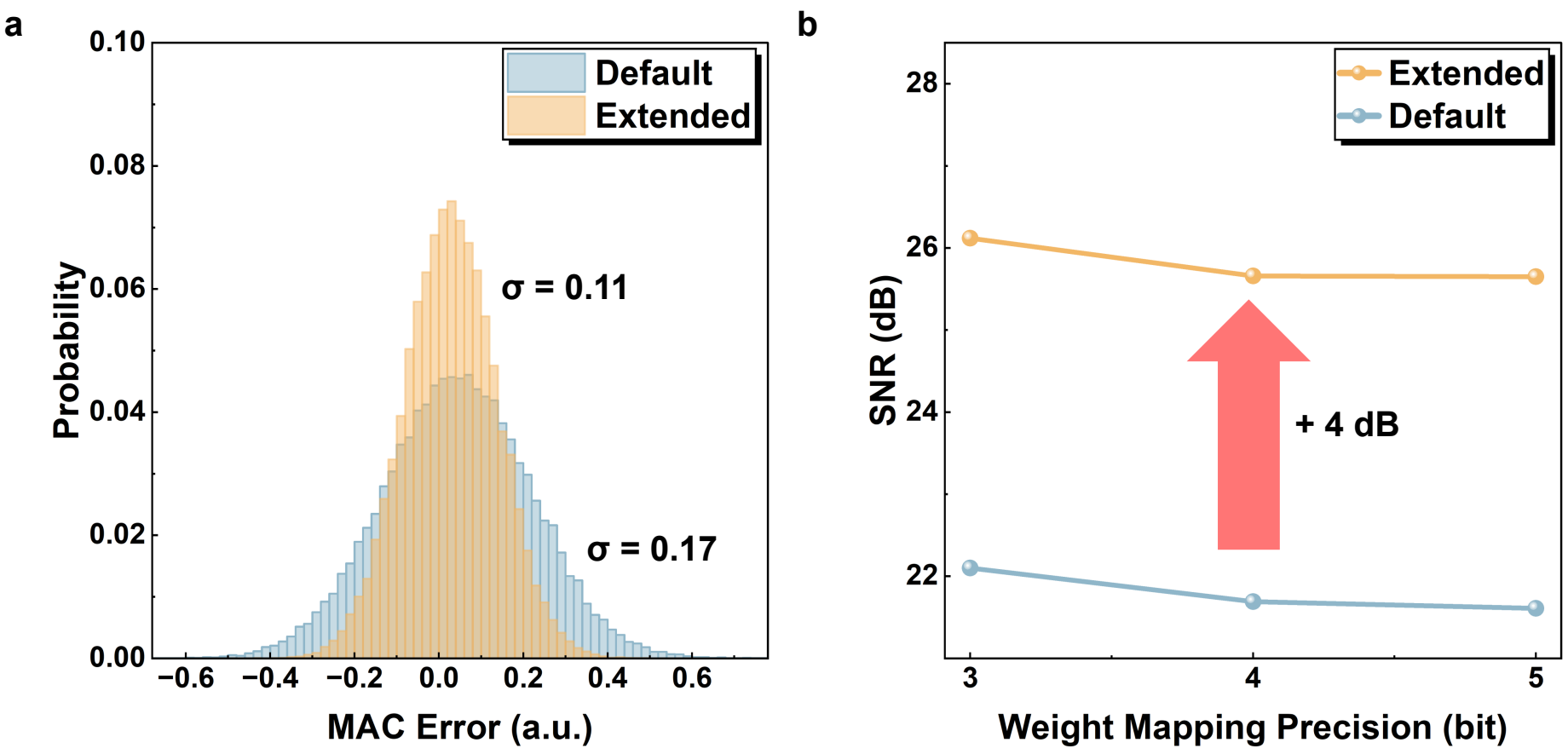

Figure S17. Variation-aware MAC robustness. (a) Distributions of the MAC error under the measured frozen log-normal variation (30 Monte-Carlo draws, 4-bit weight mapping) for the default and extended windows, showing a 1.59× reduction of the error standard deviation in the extended window. (b) MAC SNR of the default and extended windows for weight mapping precisions of 3–5 bits. The extended window improves the SNR by +4.0 dB independently of the mapping precision.

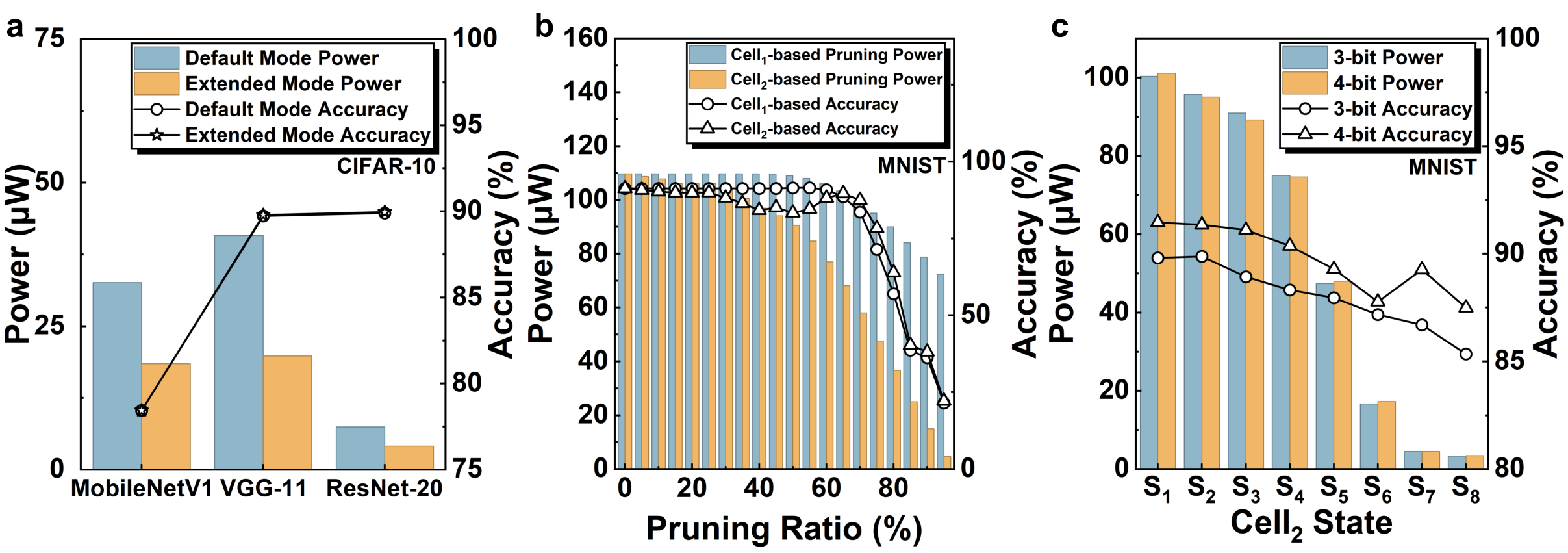

Figure S18. Monte-Carlo re-evaluation of the system-level results of Figure 7 in the main text under the measured D2D/C2C variation (20 Monte-Carlo trials per condition). Error bars indicate the standard deviation over the trials. (a) Variation-aware inference accuracy and inference power of the default and extended modes for MobileNetV1, VGG-11, and ResNet-20, compared with the software baseline (dashed lines). (b) Classification accuracy and inference power as functions of the pruning ratio, comparing minimum-conductance-state-based pruning and $Cell_2$ OFF-state-based true-off pruning. (c) Classification accuracy and inference power as functions of the $Cell_2$ state, for 3-bit and 4-bit weight quantization.

12.1 Post-pruning fine-tuning

To assess the compatibility of true-off pruning with the standard prune-then-fine-tune workflow, a post-pruning fine-tuning study was performed on the 784 × 10 MNIST classifier of Figure 7d of the main text, using the same hardware-aware evaluation pipeline as in Section 9. Weights are mapped to the 46 measured programming states of the fabricated cell through their measured current–voltage lookup tables, inputs are applied on the 51-level drain-voltage grid, and classification uses the raw hardware outputs, without any output-side correction. Both pruning implementations of Figure 7d, $Cell_2$ OFF-state-based true-off pruning and minimum-conductance-state-based pruning, were fine-tuned under identical conditions. The overall workflow of this fine-tuning study is illustrated in Figure S19. After magnitude-based pruning, only the surviving weights were fine-tuned in software, with the pruning mask kept fixed and with the same hyperparameters as the original training. When the fine-tuned weights are deployed on the true-off array, the classification accuracy at a 75% pruning ratio recovers from 78.3% (no fine-tuning) to 88.1%, close to the software fine-tuned reference of 91.0% (Figure S20a). Fine-tuning converged within a few tens of epochs, compared with 200 epochs for the original training, because only the surviving 25% of the weights are updated. In Figure S20a, the true-off accuracy falls slightly below the min-state curve at intermediate pruning ratios. The origin of this gap is that true-off pruning removes not only the weight but also the minimum-conductance current of the pruned cells, so the column-wise sum of these minimum currents is no longer identical across outputs. This term is deterministic and fully known at training time from the pruning mask and the measured conductance mapping, so it can be included in the fine-tuning forward pass, in direct analogy to standard quantization-aware training. Only the training forward pass is modified, and evaluation is still performed on the raw hardware outputs. With this hardware-aware fine-tuning (Figure S20b), the true-off array reaches 89.5% accuracy at the 75% pruning ratio, and maintains approximately 90% or higher accuracy up to a 70% pruning ratio, essentially overlapping the min-state accuracy curve while consuming less than half of its power. For the min-state implementation, hardware-aware fine-tuning brings no further improvement over standard fine-tuning (90.8% for both schemes at the 75% ratio), because pruned cells carry the same minimum-conductance current as zero-valued weights and the deployment is already matched to the software forward pass. The benefit of hardware-aware fine-tuning is specific to true-off pruning. The power advantage of true-off pruning is preserved, and even enhanced, through fine-tuning. The power benefit originates from the pruned cells, and fine-tuning never touches them. Each pruned cell conducts approximately 0.4 nA in the $Cell_2$ OFF state versus 72.7 nA in the minimum conductance state, and this per-cell current difference persists through any amount of retraining. Accordingly, after identical fine-tuning, the true-off array consumes 44.1 µW at the 75% pruning ratio whereas the min-state array consumes 91.5 µW, and even at a 95% pruning ratio the min-state power does not fall below approximately 70 µW (64% of the unpruned 109.6 µW), because every pruned cell keeps conducting the minimum current (Figure S20a,b). Moreover, fine-tuning itself provides an additional power gain. As the surviving weights are redistributed during retraining, 56% of the surviving cells move to lower current states (33% move higher), reducing their mean read current by 2.9%, so the fine-tuned true-off array consumes less power than the non-fine-tuned one at the same ratio (47.5 to 44.1 µW with standard fine-tuning, and 41.1 µW with hardware-aware fine-tuning). As a result, at a comparable fine-tuned accuracy of approximately 90.5%, true-off pruning operates at 49–58 µW, whereas minimum-conductance-state pruning requires 91.5 µW or more. The entire low-power operating region is therefore accessible only through true-off pruning.

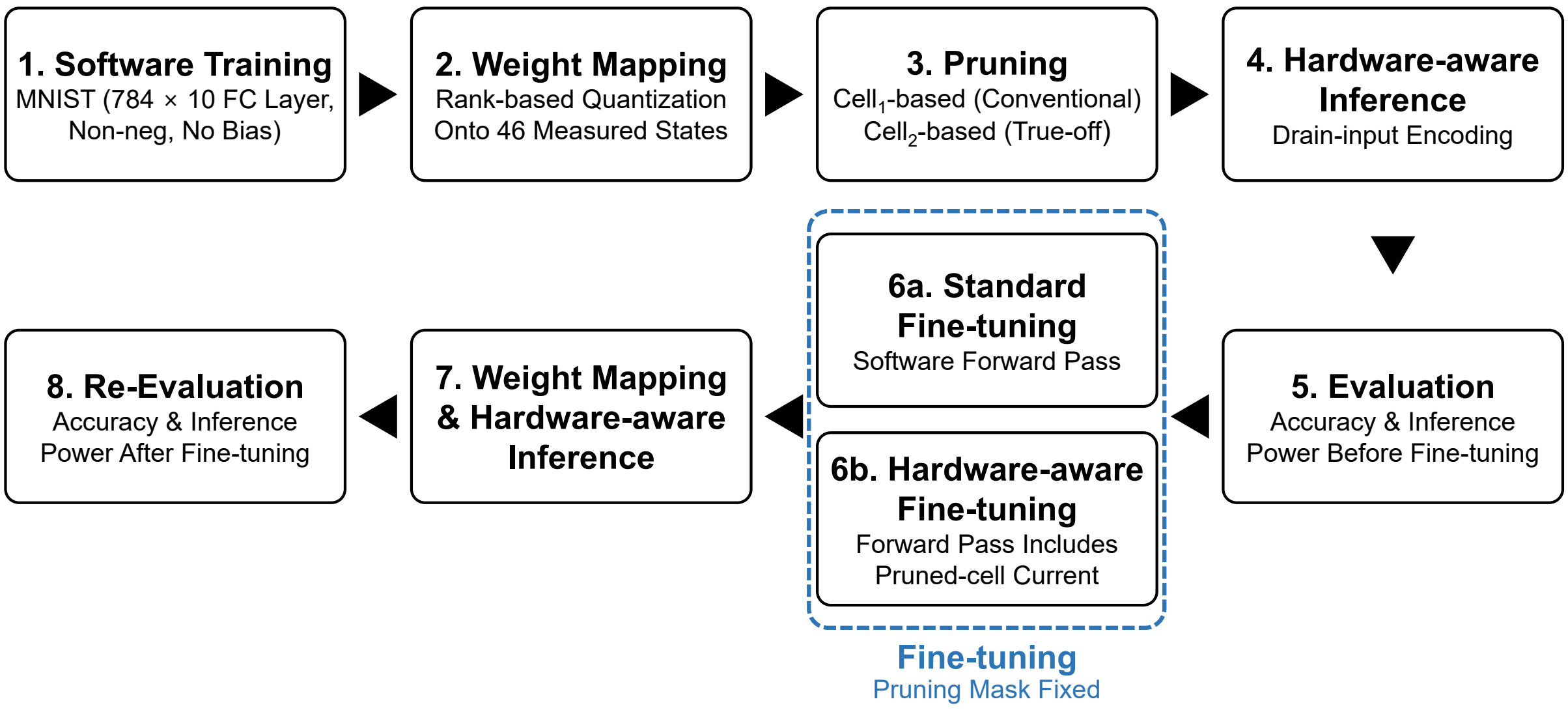

Figure S19. Workflow of the post-pruning fine-tuning study. The trained 784 × 10 MNIST classifier is mapped to the measured conductance states by rank-based quantization and pruned by either the conventional minimum-conductance-state ($Cell_1$-based) scheme or the $Cell_2$ OFF-state-based true-off scheme. After the initial hardware evaluation, the surviving weights are fine-tuned with the pruning mask kept fixed, using either standard fine-tuning (software forward pass) or hardware-aware fine-tuning in which the forward pass additionally includes the deterministic minimum-current term of the pruned cells. The fine-tuned weights are then re-mapped and re-evaluated under the identical hardware-aware pipeline (measured lookup tables of the 46 programming states, 51-level drain-input grid, raw hardware outputs, no output-side correction).

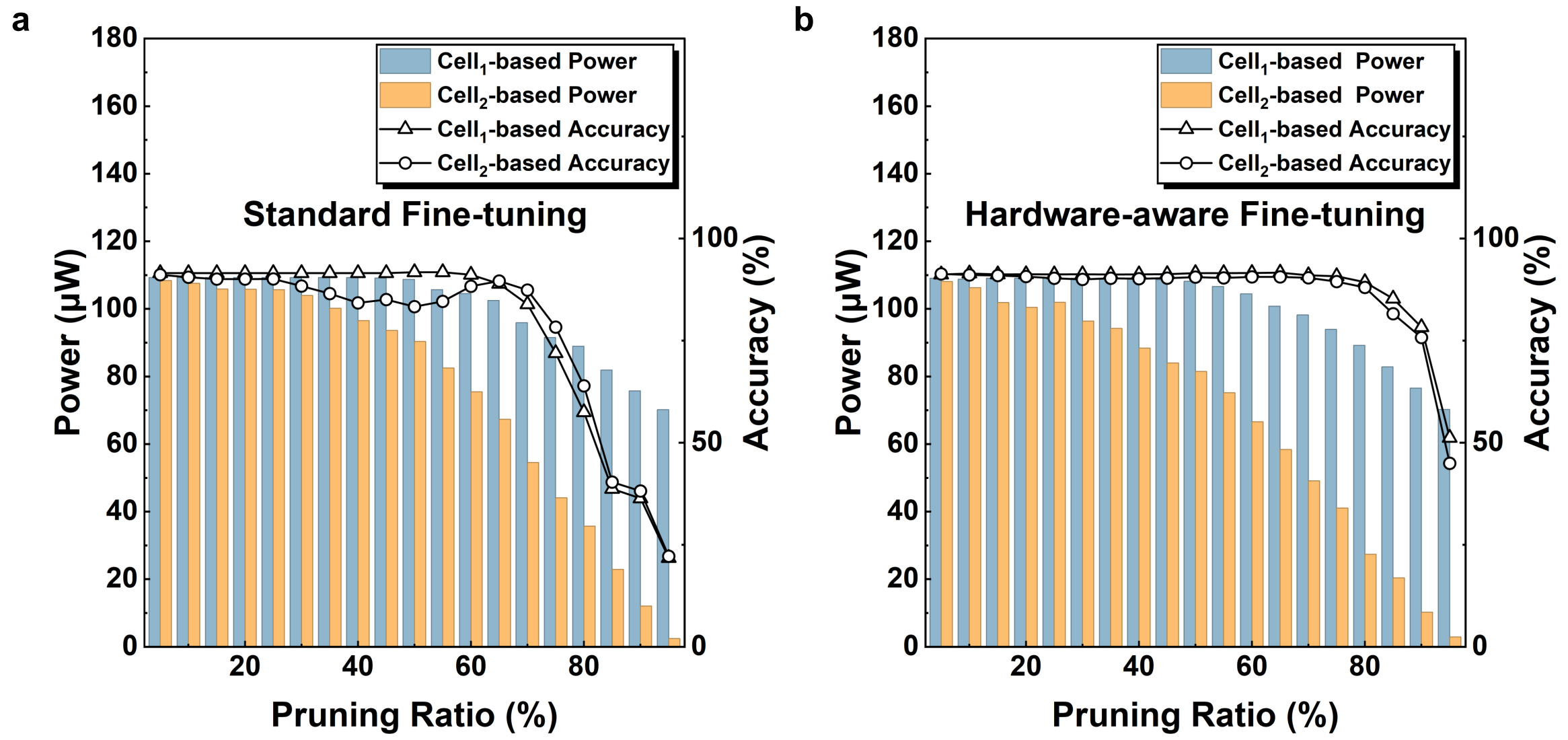

Figure S20. Post-pruning fine-tuning of the MNIST 784 × 10 classifier of Figure 7d of the main text, using the same hardware-aware evaluation pipeline (measured lookup tables of the 46 programming states, 51-level drain-input grid, raw hardware outputs, no output-side correction). Inference power (bars, left axis) and classification accuracy (open symbols, right axis) as functions of the pruning ratio, comparing Cell_2 OFF-state-based true-off pruning and minimum-conductance-state-based pruning after (a) standard software fine-tuning and (b) hardware-aware fine-tuning. The corresponding results without fine-tuning are shown in Figure 7d of the main text.

13 Extension to higher-precision signed operation

The proposed 2M architecture can be extended to 8-bit signed (INT8) weights and inputs using established multi-device techniques. Negative weights are represented by a differential pair of 2M units ($W = G^+ - G^-$), as widely adopted in analog CIM systems [18, 31]. For the weight magnitude, a single 2M unit supports 28 usable levels (≈4.8 bit) within the measurement-validated region, based on the statistical capacity analysis of Section 6. A 7-bit magnitude (128 levels) is therefore realized by bit-slicing across two units of different significance ($28^2 = 784 \gg 128$), giving a total of four 2M units per INT8 synapse (two differential branches × two slices). For the inputs, signed values are required only in limited layers in networks based on the standard ReLU activation, whose output is non-negative. However, activation functions that produce negative outputs, such as Leaky ReLU, ELU, GELU, and tanh, are also widely used. In such networks, signed inputs must be supported throughout. In both cases, the signed input can be handled by applying its positive and negative components in two sequential phases followed by digital subtraction, requiring no additional devices at a cost of 2× latency. The input resolution can be extended either by bit-serial encoding (8 cycles) or by a two-cycle decomposition of the experimentally validated 51-level input grid (2× latency for 256 levels). The area, latency, and power thus scale as approximately 4× devices per synapse and 2–8× latency depending on the input scheme, while the per-cell benefits of the extended window and true-off pruning apply unchanged to every slice. We also note that not all inference workloads require INT8 precision. The task-adaptive operation proposed in this work applies equally along the precision axis, with the precision configuration chosen purely in the weight mapping without any device modification.

14 System-level energy analysis including dynamic and peripheral components

14.1 Scope and principle

The power metric in the main text (Equation 3) captures the static, current-driven power of the synaptic array. To assess its completeness at the system level, we construct an energy ledger that additionally accounts for (i) the dynamic energy of charging and discharging the word-line, bit-line, and input-line capacitances, and (ii) the peripheral analog-to-digital converter (ADC) energy. All device-specific quantities (array currents, gate-stack composition, cell geometry) are taken from our measurements. Quantities that depend on implementation details not fabricated in this work (interconnect capacitance, ADC efficiency) are taken from the literature as ranges and swept, so that every conclusion holds across the entire plausible range rather than at a single assumed value.

14.2 Array static energy

The array energy per inference is $E_{\text{array}} = P_{\text{array}} \cdot t_{\text{read}}$, where P_{array} is the measured current-driven power (Equation 3 of the main text) and t_{read} is the read duration. The read duration is treated as a swept parameter, evaluated at two bounding scenarios, namely $t_{\text{read}} = 100$ µs, a slow-read bound at which the array static energy dominates, and $t_{\text{read}} = 100$ ns, a representative random-read access time of NOR Flash memory, at which the peripheral terms dominate.

14.3 Dynamic charging energy

The per-cell gate capacitance follows from the series combination of the measured dielectric stack, SiO_2 (4 nm, $\varepsilon_r = 3.9$) / Si_3N_4 (7 nm, $\varepsilon_r = 7.5$) / Al_2O_3 (9 nm, $\varepsilon_r = 9.0$):

$$C_{\text{area}} = \left[\sum_i t_i/(\varepsilon_0\varepsilon_{r,i})\right]^{-1} = 2.99 \text{ fF/µm}^2 \tag{3}$$

equivalent to an EOT of 11.5 nm, giving $C_{\text{WL}} = 0.75$ fF for the 0.5×0.5 µm gate. In the drain-input encoding scheme, the gate biases are DC during inference ($V_{\text{G1}} = 5$ V and $V_{\text{G2}} = 3.3$ V, held constant). The word lines are therefore charged once when inference begins, not per inference. Even this one-time charge is small. For the 784×10 array, $\frac{1}{2}C_{\text{WL}}N_{\text{cell}}V_G^2$ amounts to approximately 0.1 nJ in total, which is amortized over all subsequent inferences, and the high-impedance charge-trap gate stack draws no steady-state current. The lines that switch on every inference are the drain-side input lines (which swing with the input voltages, $\leq$1 V) and the bit lines. The capacitance per cell on a line comprises the junction and wire contributions:

$$C_{\text{line,cell}} = C_j \cdot (W \times L_{\text{diff}}) + c_w \cdot p \tag{4}$$

with the measured diffusion area $W \times L_{\text{diff}} = 0.5 \times 2.85$ µm^2 and measured bit-line pitch $p = 20$ µm. The junction capacitance $C_j = 0.5$–1.0 fF/µm^2 and wire capacitance $c_w = 0.10$–0.20 fF/µm are swept over ranges consistent with NVSim-class interconnect modeling [S1]. The per-inference dynamic energy for an $n_{\text{in}} \times n_{\text{out}}$ array is

$$E_{\text{dyn}} = \tfrac{1}{2}(C_{\text{line,cell}} \cdot n_{\text{in}})V_{\text{BL}}^2 \cdot n_{\text{out}} + \tfrac{1}{2}(C_{\text{line,cell}} \cdot n_{\text{out}})V_{\text{in}}^2 \cdot n_{\text{in}} \tag{5}$$

evaluated at the maximum swing $V = 1$ V per transition. Three deliberately conservative choices are noted. First, the row pitch of the input lines, not separately measured, is set equal to the bit-line pitch (upper bound). Second, the bit line, which is the current-summing node

held near 0 V in our readout and therefore nominally does not swing, is nonetheless assigned a full 1 V swing. Third, counting the full charge–discharge cycle energy from the supply (CV^2) instead of the per-transition $\frac{1}{2}CV^2$ merely doubles the result. Even then, E_{dyn} remains below 3% of the array static energy under the slow-read bound.

14.4 Peripheral ADC energy

The ADC energy per inference is estimated with the Walden figure of merit [S2], $E_{\text{ADC}} = N_{\text{conv}} \cdot \text{FOM}_{\text{W}} \cdot 2^B$, with one conversion per bit line ($N_{\text{conv}} = n_{\text{out}}$). The resolution B is swept from 3 to 8 bits and FOM_{W} from 1 to 100 fJ per conversion step, a range spanning the Murmann ADC performance survey [S3] from state-of-the-art to conservative designs. For $n_{\text{out}} = 10$ this yields $E_{\text{ADC}} = 0.08$–256 pJ per inference (3.2 pJ at the representative midpoint of $B = 5$ bit and 10 fJ/step). The digital accumulation and activation logic downstream of the ADC is excluded, as it is common to all compared array configurations. The steady-state current supplied by the input drivers is, by definition, the same current already counted in the array $V \cdot I$ term of Equation 3 and is therefore not double-counted.

14.5 Results

Table S7 summarizes the ledger, using the variation-mean array powers of Section 12. Under the slow-read bound ($t_{\text{read}} = 100$ µs), the array static energy accounts for 92.7–99.7% of the total system energy at the representative ADC point, and E_{dyn} remains 0.4–1.5% of E_{array} for every network. The array-level $V \cdot I$ metric of the main text therefore represents the dominant system energy component under the reported conditions. Under the NOR-Flash read timing ($t_{\text{read}} = 100$ ns), the ADC and line-charging terms become dominant, consistent with the general observation that peripheral conversion dominates analog-CIM macros at scaled operation. Because the dynamic and ADC terms depend only on the array geometry and readout precision, not on the programmed conductance states, they are identical for the default and extended configurations, and the absolute energy savings of the proposed architecture (43.3–51.5% of array energy in the extended mode, ~50% under true-off pruning, and down to below 5% under Cell_2 state modulation) are fully preserved in the system-level total.

Table S7. System-level energy ledger per inference. Array energies use the variation-mean powers (20 Monte-Carlo trials). E_{dyn} and E_{ADC} are swept over the literature ranges described in the text. The array share is evaluated at the representative ADC midpoint ($B = 5$ bit, 10 fJ/step).

Network	Config.	E_{array}@100 µs (pJ)	E_{dyn} (pJ)	E_{ADC} (pJ)	Share@100 µs	Share@100 ns
MobileNetV1 (512×10)	Default	3,258	13.9–27.8	0.08–256	99.3%	11.9%
	Extended	1,848	13.9–27.8	0.08–256	98.7%	7.1%
VGG-11 (512×10)	Default	4,081	13.9–27.8	0.08–256	99.4%	14.5%
	Extended	1,981	13.9–27.8	0.08–256	98.8%	7.6%
ResNet-20 (64×10)	Default	747	1.7–3.5	0.08–256	99.2%	11.4%
	Extended	412	1.7–3.5	0.08–256	98.6%	6.6%
MNIST (784×10)	Cell_2 state 1 (4-bit)	10,110	21.3–42.5	0.08–256	99.7%	22.4%
	Cell_2 state 7 (4-bit)	449	21.3–42.5	0.08–256	92.7%	1.3%

15 Benchmark comparison with prior CIM implementations

Table S8 compares the proposed 2M NOR Flash architecture with representative Flash-based synaptic devices and other memory-based CIM implementations along seven axes, namely cell structure, operating voltage/current, conductance resolution, inference accuracy, power/energy, peripheral overhead, and scalability. Two remarks on methodology are in order. First, the conductance resolution of this work is reported as statistically usable levels derived from the measured D2D/C2C variation with an explicit 2σ margin criterion (Section 6), which is a stricter definition than nominal level counts. Second, the compared works span different maturity levels, from fully integrated chips to device-level demonstrations with system-level evaluation (as in this work). Energy figures are annotated as measured or projected accordingly.

Table S8. Benchmark comparison with representative Flash-based synaptic devices and other memory-based CIM implementations.

	This work	[18]	[36]	[27]
Cell structure	Two-memory (2M) NOR Flash with the access transistor repurposed as a second memory device (no added device)	NOR Flash floating-gate supercell, differential pair	NAND-type charge-trap memcapacitor crossbar	Dual-gate field-effect transistor
Operating V/I	PGM/ERS 5.5–10 V (FN, ISPP), read ≤1 V, 4.22 pA–365 nA	Gate 0–4.2 V, subthreshold	Capacitive (displacement current)	Low-power operation
Conductance resolution	13 (default) → 28–39 (extended) usable levels (2σ, measured D2D/C2C)	Analog (∼5%/cell)	Analog capacitance states	Analog multi-level
Inference accuracy	MNIST 91.5%, CIFAR-10 78.5–90.3% (maintained under measured variation)	MNIST 94.7% (on-chip)	CIFAR-10 92.1% (simulation)	On-chip trainable NN
Power/energy	0.36–1.3 fJ/MAC (array, @100 ns projected), −41.7–50.2% (extended), −50% (pruning), <5% (Cell_2 modulation)	∼20 nJ per classification, <1 µs	Ultra-low static (capacitive)	Low-power
Peripheral overhead	Swept system ledger (WL static, dynamic <1.5% of array E, ADC Walden 3–8 bit)	On-chip neurons, shift register	Charge-based sensing	–
Scalability	Standard FN NOR-Flash flow, no extra masks, INT8 path with 4 units/synapse	Embedded 180 nm (55 nm projected)	NAND-structure compatible	Si CMOS-compatible

	This work	[9]	[33]	[31]
Cell structure	Two-memory (2M) NOR Flash with the access transistor repurposed as a second memory device (no added device)	One-transistor–one-resistor (1T–1R) RRAM cell (NeuRRAM), differential pair	One-transistor–one-resistor (1T–1R) RRAM cell	Phase-change memory (PCM), two PCM devices with a three-transistor–one-capacitor (3T–1C) unit per synapse
Operating V/I	PGM/ERS 5.5–10 V (FN, ISPP), read ≤1 V, 4.22 pA–365 nA	SET/RESET 1.2/1.5 V, conductance 1–40 µS	Read 0.2 V	PCM programming pulses
Conductance resolution	13 (default) → 28–39 (extended) usable levels (2σ, measured D2D/C2C)	∼4-bit equivalent	32 levels	Multi-significance pairs
Inference accuracy	MNIST 91.5%, CIFAR-10 78.5–90.3% (maintained under measured variation)	MNIST 99.0%, CIFAR-10 85.7%	MNIST >96%	Software-equivalent accuracy
Power/energy	0.36–1.3 fJ/MAC (array, @100 ns projected), −41.7–50.2% (extended), −50% (pruning), <5% (Cell_2 modulation)	2–3× lower EDP vs prior RRAM-CIM	$\sim 10^2\times$ vs GPU	28,065 GOPS/W (projected)
Peripheral overhead	Swept system ledger (WL static, dynamic <1.5% of array E, ADC Walden 3–8 bit)	Voltage-mode neurons, 1–8 b in / 1–10 b out	Off-chip peripheral system	Projected
Scalability	Standard FN NOR-Flash flow, no extra masks, INT8 path with 4 units/synapse	Monolithic 130 nm, 3M cells	Multi-array demonstrated	Mixed hardware–software demonstration

Abbreviations: PGM/ERS = program/erase, FN = Fowler–Nordheim tunneling, ISPP = incremental step pulse programming, D2D/C2C = device-to-device/cycle-to-cycle variation, WL = word line, ADC = analog-to-digital converter, EDP = energy–delay product.

Supporting References

[S1] X. Dong, C. Xu, Y. Xie, N. P. Jouppi, IEEE Trans. Comput.-Aided Des. Integr. Circuits Syst. 2012, 31, 994.

[S2] R. H. Walden, IEEE J. Sel. Areas Commun. 1999, 17, 539.

[S3] B. Murmann, ADC Performance Survey 1997–2023, available online at `github.com/bmurmann/ADC-survey`.
